# Astroglial dysfunctions drive aberrant synaptogenesis and social behavioral deficits in mice with neonatal exposure to lengthy general anesthesia

**DOI:** 10.1371/journal.pbio.3000086

**Published:** 2019-08-21

**Authors:** Bin Zhou, Lingmin Chen, Ping Liao, Lu Huang, Zhuo Chen, Daqing Liao, Linghui Yang, Jing Wang, Guoqiang Yu, Li Wang, Jianguo Zhang, Yunxia Zuo, Jin Liu, Ruotian Jiang

**Affiliations:** 1 Laboratory of Anesthesia and Critical Care Medicine, Sichuan University, Chengdu, Sichuan, China; 2 Translational Neuroscience Center, Sichuan University, Chengdu, Sichuan, China; 3 Department of Anesthesiology of West China Hospital, Sichuan University, Chengdu, Sichuan, China; 4 Bradley Department of Electrical & Computer Engineering, Virginia Polytechnic Institute and State University, Arlington, Virginia, United States of America; 5 Center for Biological Imaging, Institute of Biophysics, Chinese Academy of Sciences, Beijing, China; The Johns Hopkins University School of Medicine, UNITED STATES

## Abstract

Lengthy use of general anesthetics (GAs) causes neurobehavioral deficits in the developing brain, which has raised significant clinical concerns such that the United States Food and Drug Administration (FDA) is warning on the use of GAs in children younger than 3 years. However, the molecular and cellular mechanisms for GAs-induced neurotoxicity remain largely unknown. Here, we report that sevoflurane (Sevo), a commonly used GA in pediatrics, caused compromised astrocyte morphogenesis spatiotemporally correlated to synaptic overgrowth, with reduced synaptic function in developing cortex in a regional-, exposure-length-, and age-specific manner. Sevo disrupted astrocyte Ca^2+^ homeostasis both acutely and chronically, which led to the down-regulation of Ezrin, an actin-binding membrane-bound protein, which we found was critically involved in astrocyte morphogenesis in vivo. Importantly, overexpression of astrocyte Ezrin rescued astrocytic and neuronal dysfunctions and fully corrected deficits in social behaviors in developing mice with lengthy Sevo exposure. Our data uncover that, in addition to neurons, astrocytes may represent important targets for GAs to exert toxic effects and that astrocyte morphological integrity is crucial for synaptogenesis and neurological behaviors.

## Abstract 2

The extended use of general anesthetics can cause neurobehavioral deficits in the developing brain, leading to clinical concerns regarding their use in children younger than 3 years. This study shows that general anesthetics target glial cells to disrupt neural circuit formation in the developing brain, an effect that may underlie the observed learning, cognitive, or emotional deficits.

## Introduction

Millions of infants and young children are undergoing general anesthesia every year. Human retrospective cohort studies strongly suggest that exposure of general anesthetics (GAs) and sedation drugs to immature individuals produced developmental and behavioral disorders and learning disabilities [1,2]. More recently, the high-profile clinical trials, the General Anaesthesia compared to Spinal anaesthesia (GAS) [3,4], Pediatric Anesthesia Neurodevelopment Assessment (PANDA) [5], and Mayo Anesthesia Safety in Kids (MASK) studies [6] found that single short exposures of GAs did not produce aberrant neurologic behaviors. However, the MASK study also suggested that multiple GAs exposure (mean duration of anesthesia, 4.9 h) is associated with decreases in processing speed and fine motor coordination. The anesthetic-induced developmental neurotoxicity (AIDN) has raised significant concerns to clinicians, scientists, and the public about the safety of general anesthesia to infants and young children. In light of the current clinical and preclinical evidence, in 2016, the US Food and Drug Administration (FDA) issued a drug safety communication warning that “repeated or lengthy use of general anesthetic and sedation drugs in children younger than 3 years or in pregnant women may affect the development of children’s brains” (https://www.fda.gov/Drugs/DrugSafety/ucm532356.htm). In summary, the existing clinical evidence is sufficiently concerning that more efforts are required to elucidate the mechanisms by which GAs exposure, particularly lengthy or repeated exposure, affects brain development.

Preclinical studies have clearly shown that early lengthy/repeated exposure of nearly all commonly used GAs, including sevoflurane (Sevo), isoflurane, propofol, and ketamine, cause neurobehavioral disorders in the developing rodent brain [1,2]. These disorders include spatial [1], no-spatial [7], and fear condition learning deficits [8] in rodents and anxiety-related behaviors or motor reflex deficits in nonhuman primates [2]. However, the underlying molecular and cellular mechanisms are not completely understood. Early studies mainly focused on the apoptotic hypothesis. Nevertheless, in most studies, only a very small fraction of cells displayed activated caspase-3 expression after GAs exposure, and there is no strong evidence for a causal link between neuronal apoptosis and lasting neurocognitive impairment [2]. Recently, it has been shown that early lengthy exposure of GAs induced increased or decreased dendrite outgrowth, disruption of axon guidance, and altered synaptic functions in mouse cortex and hippocampus [2,7,9,10]. Aberrant synaptogenesis and neural circuit formation have strong causal links with many psychiatric and neurodevelopmental disorders. However, little is known regarding the molecular and cellular mechanisms underlying the synaptic and circuit dysfunctions induced by GAs during the critical period of the brain development.

Astrocytes constitute at least one third of human glial cells. Astrocyte processes contact with axons and dendritic spines, forming “tripartite synapses,” and play vital roles in the process of synaptogenesis during brain development [11,12]. Sparse studies from the literature suggest that lengthy GAs exposure altered astrocyte functions in vitro, including impaired expressions of glial fibrillary acidic protein (GFAP), glutamate-aspartate transporter (GLAST) and brain-derived neurotrophic factor (BDNF) [13,14], and astrocyte capability to support neuronal development [15]. However, most of the studies described above either used in vitro exposure to cultured astrocytes, which differs significantly from general anesthesia in vivo, or provided little information on how astrocyte dysfunctions correlated or contributed to measurable neuronal deficits under the same experimental settings. Hence, how GAs affect astrocyte developmental biology and how GAs disrupt glia-neuron interactions during synaptogenesis in vivo remain largely unexplored.

In the current study, in a combination of Ca^2+^ imaging, high-resolution morphological reconstructions using light and electron microscopy, and mouse genetics, we explored in detail how Sevo, a commonly used GA in pediatrics, negatively impacts astrocyte morphogenesis and Ca^2+^ signaling in situ and in vivo, and provided evidence to show that astrocyte dysfunctions drive neuronal dysfunctions and social behavior deficits in mice with lengthy Sevo exposure.

## Results

### Lengthy Sevo exposure disrupts astrocyte morphogenesis with compromised “tripartite synapse” maturation in developing somatosensory cortex

To evaluate how lengthy Sevo exposure affects developing mouse brain, we had postnatal day 7 (P7) mice exposed to 2.5% Sevo (termed hereafter Sevo group mice) or their littermates exposed to carrier gas (30% O_2_/70% N_2_) (termed hereafter Control group mice) for 4 h. This experimental setting was chosen based on a number of previous preclinical studies in rodents and nonhuman primates [16–19], clinical observations [20], as well as the FDA warning. All mice survived after exposure, with both artery blood gas parameters measured immediately after exposure (**S1 Table**) and body weight at P14 comparable to the Control group mice (Control group versus Sevo group: 6.55 ± 0.73 g versus 6.27 ± 0.73 g, *P* = 0.299, *n* = 15 and *n* = 17 mice in Control and Sevo group, respectively, unpaired *t* test).

**Fig 1:**
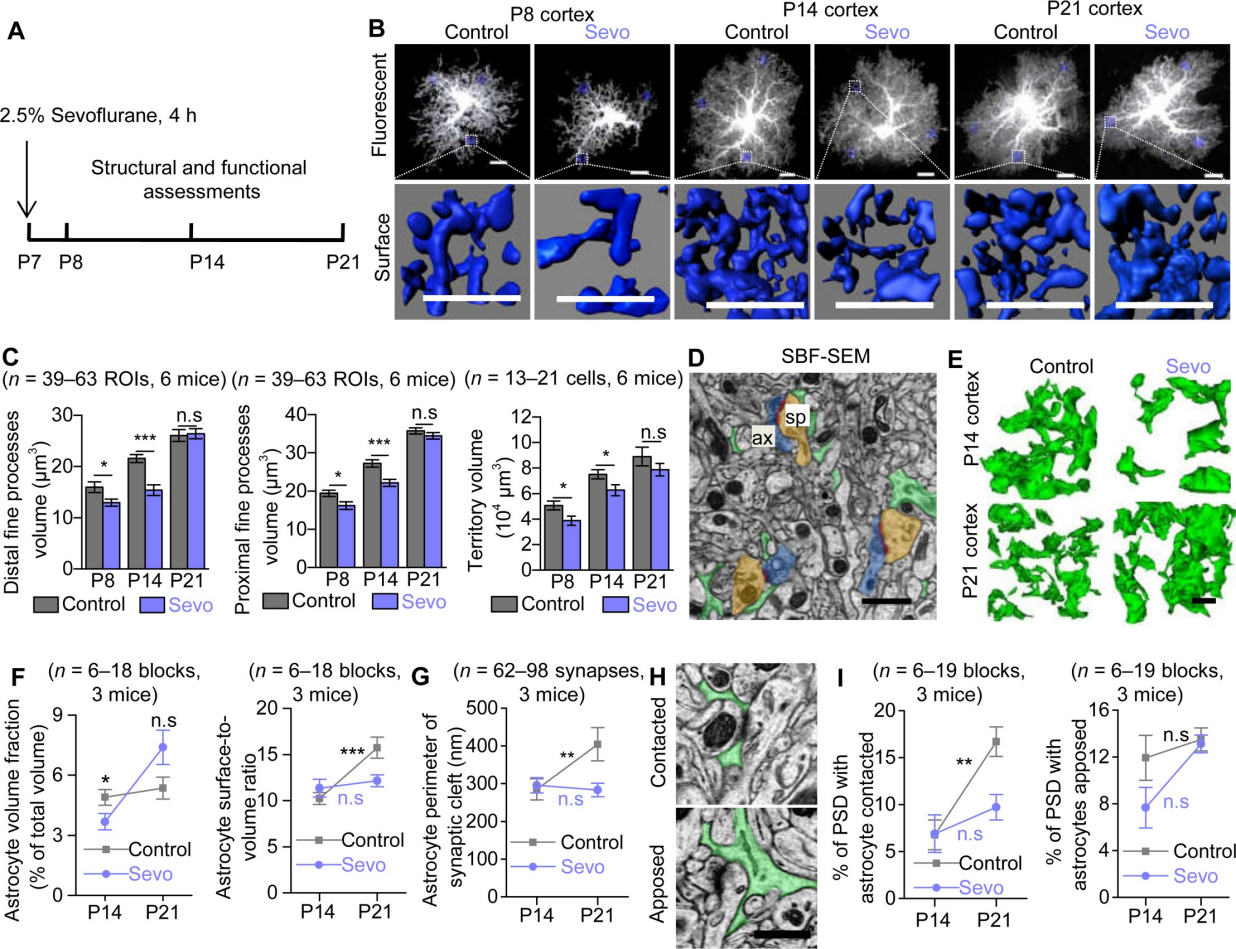
Lengthy Sevo exposure disrupted astrocyte morphogenesis and compromised “tripartite synapse” maturation in developing somatosensory cortex. (**A**) Schematic illustrating the experimental protocol for Sevo exposure. (**B**) Representative confocal images and 3D reconstructed distal fine processes of sparsely labeled astrocytes from Control and Sevo group mice at P8, P14, and P21. Scale bars, 10 μm. (**C**) Quantification of astrocyte distal fine processes volume (**Left**) (P8: *P =* 0.02; P14: *P* < 0.001; P21: *P* = 0.82; unpaired *t* test), proximal fine processes volume (**Middle**) (P8: *P =* 0.012; P14: *P* < 0.001; P21: *P* = 0.279; unpaired *t* test), and territory volume (**Right**) (P8: *P =* 0.028; P14: *P* = 0.03; P21: *P* = 0.248; unpaired *t* test) in Control and Sevo mice. (**D**) SBF-SEM images of astrocyte fine processes (green), PSD (red), axonal bouton (ax, blue), and dendritic spine (sp, brown). Scale bar, 1 μm. (**E**) Three-dimensional reconstruction of astrocyte fine processes in Control and Sevo group mice at P14 and P21. Scale bar, 1 μm. (**F**) **Left**, comparison of astrocyte volume fraction in Control and Sevo group mice (P14: *P* = 0.038; P21: *P* = 0.074; unpaired *t* test). **Right**, increased astrocyte surface-to-volume ratio from P14 to P21 in Control (*P* < 0.001, unpaired *t* test) group mice but not in Sevo group mice (*P* = 0.628, unpaired *t* test). (**G**) Increased astrocyte perimeter enwrapping the synaptic cleft from P14 to P21 in Control (*P* = 0.007, Mann-Whitney test) group mice but not in Sevo group mice (*P* = 0.232, Mann-Whitney test). (**H**) SBF-SEM images of astrocytes contacted or apposed PSD. Scale bar, 0.5 μm. (**I**) **Left**, increased PSD with astrocytes contacted from P14 to P21 in Control group mice (*P* = 0.002, Mann-Whitney test) but not in Sevo group mice (*P* = 0.129, Mann-Whitney test). **Right**, unchanged PSD with astrocytes apposed from P14 to P21 in Control (*P* = 0.105, Mann-Whitney test) and Sevo (*P* = 0.07, unpaired *t* test) groups. *P < 0.05; **P < 0.01; ***P < 0.001; n.s, not significant. Data are shown as mean ± SEM. Underlying data are available in S1 Data. n.s, not significant; PSD, postsynaptic density; SBF-SEM, serial block face scanning electron microscopy; Sevo, sevoflurane.

Then, we examined in detail how lengthy Sevo exposure affected astrocyte morphogenesis during the critical period of the brain development, i.e., from P7 to P21 in mice [12]. To achieve this, we performed intracellular lucifer yellow iontophoresis in lightly fixed tissue followed by confocal imaging and morphological 3D reconstructions to analyze astrocyte morphology in situ [21] at P8, P14, and P21 (**Fig 1A**). In somatosensory cortex, astrocytes from Sevo group mice displayed significantly smaller distal (away from soma) and proximal (closed to soma) fine process volume, with decreased territory volume, measured at both P8 and P14. However, when at P21, there was no more significance between the two groups (**Fig 1B** and **1C**). No significant difference was observed between the two groups in astrocyte soma and primary branches (branches directly protruding from soma) at P8, P14, and P21 (**S1A** and **S1B Fig**). We also quantified GFAP expressions using immunostaining, a well-known marker for astrogliosis, and no significant difference between the two groups was found (**S1C** and **S1D Fig**). Therefore, our data so far suggest that Sevo exposure induced compromised astrocyte fine structure development without causing apparent astrogliosis in the mouse cortex.

In contrast to the cortex, much fewer morphological deficits were observed in the CA1 stratum radiatum (CA1sr) or in the molecular layer of dentate gyrus (DG-mo) of the hippocampus at P14 (**S2 Fig**), a time point when cortical astrocytes showed remarkable morphological deficits in Sevo group mice. In clinical studies, cognitive dysfunctions are mainly associated with early childhood exposure to lengthy general anesthesia. We also tested if developing astrocytes were resistant to a relatively short Sevo exposure and if mature astrocytes were resistant to a 4-h Sevo exposure. Interestingly, neither cortical astrocytes in P7 mice with only 1 h Sevo exposure measured at P14 nor those in adult mice (approximately P45) with 4-h Sevo exposure measured 12 h later (**S3 Fig**) displayed morphological deficits. Together, our data reveal that lengthy but not short Sevo exposure induces compromised astrocyte morphogenesis only in developing but not in mature brain.

Astrocyte processes are extremely fine (<50 nm) and form “tripartite synapses” with axonal boutons and dendritic spines [22]. We then performed serial block face scanning electron microscopy (SBF-SEM) to examine the ultrastructure of the tripartite synapse. Lower astrocyte volume fraction (which reflects the absolute volume of astrocyte processes) was observed in the somatosensory cortex of Sevo group mice at P14, but not at P21. From P14 to P21, the surface-to-volume ratio (which reflects the fineness of astrocyte processes) was markedly increased by approximately 50% in Control group mice but remained unchanged in Sevo group mice (**Fig 1D–1F**). Then we examined astrocyte-neuron contact. There was also an increase of astrocyte perimeters enwrapping the synaptic cleft by approximately 30% from P14 to P21 in Control group mice, but remained unchanged in Sevo group mice (**Fig 1G**). In addition, there was also increase of astrocyte fine processes insertion into synaptic cleft from P14 to P21 by approximately 147%, as shown by the markedly increased number of astrocyte-contacted postsynaptic density (PSD) in Control group mice, but remained unchanged in Sevo group mice. The number of astrocyte-apposed PSD remained unchanged in both groups from P14 to P21 (**Fig 1H** and **1I**).

The light and electron microscopic data together demonstrate that lengthy Sevo exposure disrupts astrocyte morphogenesis, resulting in altered tripartite synaptic structure in a regional-, exposure-length-, and age-specific manner.

### Aberrant synaptic growth and functions spatiotemporally correlated with astrocyte morphological deficits in Sevo group mice

**Fig 2:**
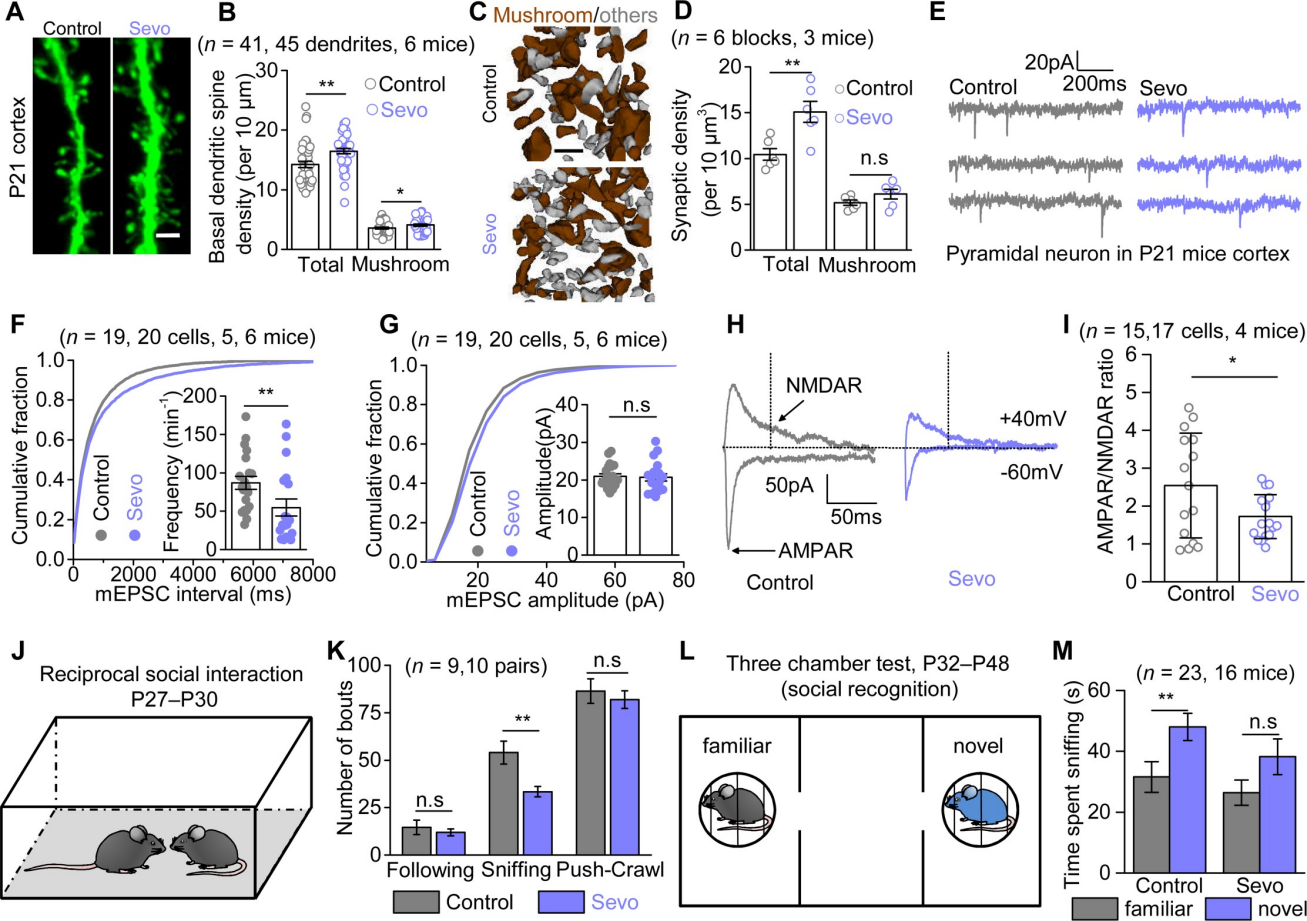
Aberrant synaptic growth and function along with disrupted social interactions in Sevo group mice. (**A**) Representative images of basal dendritic spines in the somatosensory cortex of Control and Sevo groups at P21. Scale bar, 2 μm. (**B**) Quantification of the total (*P* < 0.001, unpaired *t* test) and mushroom (*P* = 0.039, unpaired *t* test) dendritic spine density in Control and Sevo groups at P21. (**C**) Three-dimensional reconstruction of mushroom spines (brown) and other morphologically immature spines (gray). Scale bar, 1 μm. (**D**) Quantification of total (*P* = 0.005, unpaired *t* test) and mushroom (*P* = 0.155, unpaired *t* test) synaptic density in mice of Control and Sevo groups. (**E**) Representative mEPSCs traces from L3-5 pyramidal neurons in the somatosensory cortex. **(F**) Cumulative distributions and average frequency (*P* = 0.007, Mann-Whitney test) of mEPSCs. (**G**) Cumulative distributions and average (*P* = 0.833, unpaired *t* test) of mEPSCs amplitude. (**H**) Representative eEPSCs traces from L3-5 pyramidal neurons from Control and Sevo mice at P21. The black dotted line (50 ms after the stimulation) indicated the measuring points for NMDAR-eEPSCs amplitudes. (**I**) Quantification of AMPAR/NMDAR ratio (*P* = 0.033, unpaired *t* test) from Control and Sevo groups. (**J**) Cartoon illustration for reciprocal social interaction test. (**K**) Quantification of the reciprocal social interaction test (following: *P* = 0.039, Mann-Whitney test; sniffing: *P* = 0.005; push-crawl: *P* = 0.578; unpaired *t* test) in pairing mice from Control and Sevo group. (**L**) Cartoon illustration for three-chamber test (social recognition). (**M**) Quantification of the social recognition test in mice of Control and Sevo groups (Control: *P* = 0.008; Sevo: *P* = 0.086; Mann-Whitney test). *P < 0.05; **P < 0.01; n.s, not significant. Data are shown as mean ± SEM. Underlying data are available in S1 Data. AMPAR, α-amino-3-hydroxy-5-methyl-4-isoxazole propionate receptor; eEPSC, evoked excitatory postsynaptic current; L3-5, layer 3–5; mEPSC, miniature excitatory postsynaptic current; NMDAR, N-methyl-D-aspartic acid receptor; n.s, not significant.

Lengthy Sevo exposure has been reported to increase apoptosis [23]. However, in our experimental paradigm, no marked apoptosis was found in Sevo mice at P8 (**S4A–S4D Fig**), compared with the Control mice (<1% in both groups). Because there is no strong evidence for a causal link between apoptosis and lasting neurobehavioral impairment [2], we did not study the apoptosis in depth; instead, we focused on whether lengthy Sevo exposure produces structural and functional deficits in cortical pyramidal neurons at a later stage (P21). By performing lucifer yellow iontophoresis in combination with morphological 3D reconstruction, we found that the cortical pyramidal neurons in Sevo group mice had higher total and mushroom basal dendritic spine density than those in Control group mice at P21 (**Fig 2A and 2B**). Interestingly, the dendritic spine density was similar in the hippocampal CA1sr (**S4E** and **S4F Fig**) between the two groups, recalling the unchanged astrocyte morphology in this region. Using SBF-SEM, we identified structurally visible PSDs and reconstructed the corresponding dendritic spines as indictors for excitatory synapses (**Fig 2C**). We found a more than 50% higher total synaptic density, whereas the mushroom synaptic density remained unchanged in the primary somatosensory cortex in Sevo group mice (**Fig 2D**). This dataset suggests that the synaptic overgrowth was present in parallel with astrocytic structural deficits in the cortex but not in the hippocampus of Sevo group mice, and SBF-SEM data suggest the synaptic overgrowth largely resulted from an increase of synapses that are relatively immature or less functional.

Was the synaptic overgrowth associated with enhanced synaptic function? To address this question, we measured α-amino-3-hydroxy-5-methyl-4-isoxazole propionate receptor (AMPAR)-mediated miniature excitatory postsynaptic currents (mEPSCs) from pyramidal neurons in the cortical layer 3–5 (L3-5) using the whole cell patch-clamp technique (**Fig 2E**). Unexpectedly, the frequency of mEPSCs was not increased but instead markedly decreased by approximately 37% in the Sevo group mice compared with the Control group mice (**Fig 2F**), whereas the amplitudes of mEPSCs were similar (**Fig 2G**). This dataset suggests that Sevo exposure resulted in a decrease of glutamate release probability or (and) a decrease in the number of AMPAR-containing synapses. Then we measured the evoked EPSCs (eEPSCs) in pyramidal neurons (**Fig 2H**). We confirmed that the evoked currents were truly AMPAR- and N-methyl-D-aspartic acid receptor (NMDAR)-mediated currents by using AMPAR- and NMDAR-specific antagonists 6-cyano-7-nitroquinoxaline-2,3-dione (CNQX) and DL-2-Amino-5-phosphonopentanoic acid (DL-AP5), respectively (**S5A Fig**). We found that the AMPAR/NMDAR ratio was significantly approximately 33% smaller in the Sevo group when compared with the Control group (**Fig 2H and 2I**), with unchanged decay kinetics and input resistance (**S5B–S5D Fig**). Because the reduction (37%) in mEPSCs frequency can quantitatively account for the reduction in AMPAR/NMDAR ratio (33%) observed in Sevo group mice, these results suggest that the synaptic NMDAR function is largely unaffected in these mice. The structural and functional data together reveal that early Sevo exposure induces an increase in synapse number, some of which are very likely nonfunctional/immature, with reduced AMPAR-mediated synaptic transmission in the cortex, where astrocytes display compromised morphogenesis.

### Behavioral deficits in Sevo group mice

Neonatal lengthy Sevo exposure was found to produce abnormal neurological behaviors including cognitive impairment and social behavior deficits in the adulthood [8,17,24]. We first assessed the hippocampus-associated cognitive functions in the two group mice. In line with no marked astrocyte or neuron morphological deficits identified in hippocampus, we found no difference in the two group mice in the Morris water maze and fear conditioning tests (P42–P57) (**S6A** and **S6B Fig**). We then evaluated the cortex-related social behaviors using the reciprocal social interaction test (P27–P30) and three-chamber test (P32–P48) [25]. In the reciprocal social interaction test, the Sevo mice showed significantly fewer sniffing behaviors, with no difference in following and push-crawl behaviors as compared with the Control mice, suggesting less social interaction in the Sevo group mice (**Fig 2J** and **2K**). We further assessed sociability and social recognition by using the three-chamber test. In the sociability paradigm, we tested whether mice preferred a social stimulus (mouse) to no-social stimulus (empty). Both the Control and Sevo group mice showed a strong preference for the social stimulus, as they spent more time sniffing the mouse than the empty cage (**S6C Fig**). Therefore, the sociability was unaltered in Sevo mice. Next, we assessed social recognition by testing whether subject mice preferred a novel stimulus to a familiar stimulus (**Fig 2L**). As expected, the Control group mice showed a strong preference for a novel stimulus. By contrast, the Sevo group mice did not show preference for a novel stimulus over a familiar stimulus, suggesting compromised social recognition in the Sevo group mice (**Fig 2M**). No difference in spontaneous home cage activity, rotarod, or open field test was observed between the two group of mice, suggesting the social behavioral deficits in the Sevo group mice were not due to compromised locomotive ability (**S6D–S6F Fig**).

### Sevo exposure disrupted Ca^2+^ signals in developing cortical astrocytes

**Fig 3:**
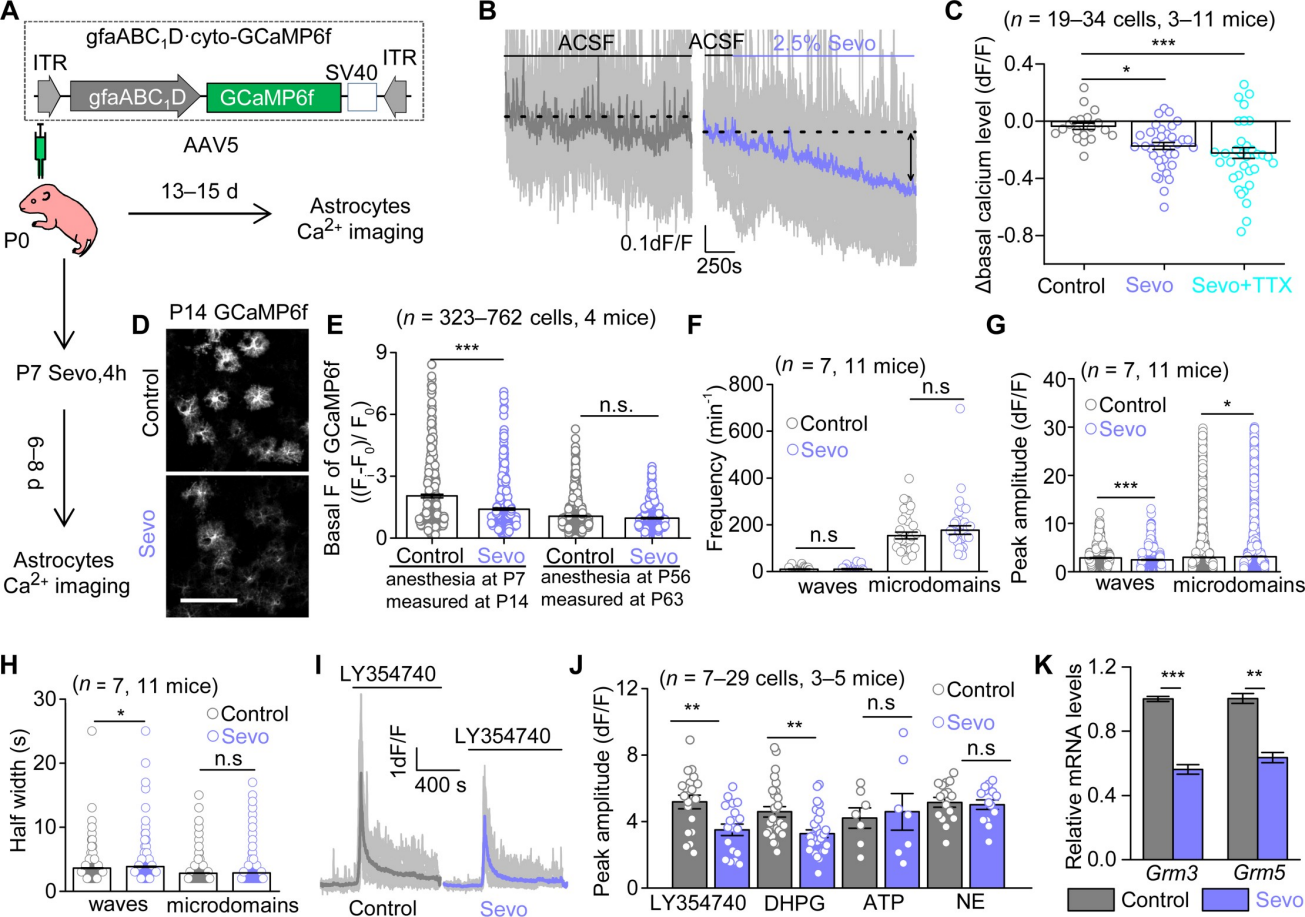
Sevo exposure disrupts Ca^2+^ signals in developing cortical astrocytes. (**A**) Schematic of Ca^2+^ imaging protocols. (**B**) Individual traces (light gray) and average trace (purple) showing altered astrocyte basal Ca^2+^ level by Sevo exposure. (**C**) Quantification of the effect of Sevo or Sevo + TTX (Sevo: *P* = 0.015; Sevo + TTX: *P* < 0.001; one-way ANOVA followed by post hoc Tukey test) on cortical astrocyte basal Ca^2+^ levels. (**D**) Representative confocal images of GCaMP6f-expressing astrocytes. Scale bar, 100 μm. (**E**) Quantification of basal fluorescent intensity of GCaMP6f (P14: *P* < 0.001; P56: *P* = 0.523; Mann-Whitney test), F_i_: fluorescent intensity of GCaMP6f-expressing cells; F_0_: background intensity. (**F, G, H**) Quantification of frequency, peak amplitude, and half-width for the two types of spontaneous Ca^2+^ signals at P13–P15 (*P* = 0.672 for waves frequency; *P* = 0.314 for microdomains frequency; *P* < 0.001 for waves peak amplitude; *P* = 0.037 for microdomains peak amplitude; *P* = 0.012 for waves half-width; *P* = 0.771 for microdomains half-width, Mann-Whitney test). (**I**) Individual traces (light gray) and averages for Ca^2+^ responses to LY354740 (10 μM) at P13–P15. (**J**) Summary bar graph for peak amplitude of Ca^2+^ signals evoked by LY354740 (*P* = 0.004, unpaired *t* test), DHPG (100 μM) (*P* = 0.001, Mann-Whitney test), ATP (300 μM) (*P* = 0.770, unpaired *t* test), and NE (20 μM) (*P* = 0.730, unpaired *t* test). (**K**) Quantification of relative mRNA levels of mGluR3 (*P* < 0.001, unpaired *t* test) and mGluR5 (*P* = 0.0018, unpaired *t* test) by RT-qPCR analyzing of GLAST^+^ MACS cells (*n* = 3 independent preparations). *P < 0.05; **P < 0.01; ***P < 0.001; n.s, not significant. Data are shown as mean ± SEM. Underlying data are available in S1 Data. DHPG, dihydroxyphenylglycine; GCaMP6f, AAV5•gfaABC_1_D•GCaMP6f; GLAST, glutamate-aspartate transporter; *Grm3* or mGluR3, metabotropic glutamate receptor 3; *Grm5* or mGluR5, metabotropic glutamate receptor 5; ITR, inverted terminal repeat; LY354740, (1S,2S,5R,6S)-2-Aminobicyclo[3.1.0]hexane-2,6-dicarboxylic acid; MACS, magnetic cell sorting; NE, noradrenaline; n.s, not significant; RT-qPCR, quantitative reverse transcription PCR; Sevo, sevoflurane; TTX, tetrodotoxin.

Next, we performed a mechanistic study to reveal why the astrocyte morphogenesis was compromised in Sevo group mice. Thrane and colleagues reported that GAs (ketamine/xylazine, isoflurane, and urethane) exposure suppressed acutely spontaneous Ca^2+^ signals and evoked Ca^2+^ responses in vivo in mature astrocytes [26], which prompted us to measure the acute and chronic effects of lengthy Sevo exposure on developing cortical astrocytes. AAV5·gfaABC_1_D·GCaMP6f (GCaMP6f) was expressed in cortical astrocytes using in vivo microinjections at P0 (**Fig 3A**) (see Materials and methods). GCaMP6f microinjections at P0 did not induce apparent astrogliosis (**S7 Fig**) at P14.

The assessment of acute effects of Sevo on astrocyte Ca^2+^ signals was achieved by continuous infusion of Sevo (0.36 ± 0.03 mM), equivalent to 2.5% Sevo [27], in acute brain slices from wild-type (WT) mice at P14. Interestingly, we found that acute Sevo exposure led to a gradual and persistent loss of basal Ca^2+^ level in cortical developing astrocytes, and the effect was not driven by action potential firing, as basal Ca^2+^ level still decreased in the presence of 300 nM tetrodotoxin (TTX) (**Fig 3B** and **3C**). Independently, we performed GCaMP6f microinjections at P0, Sevo/Control exposure in vivo at P7, and we found that the GCaMP6f basal fluorescent intensity in Sevo group mice was significantly lower than in the Control group mice (**Fig 3D and 3E**) when measured at P14, suggesting that the loss of basal Ca^2+^ level was not fully recovered within 6–8 d after exposure. To explore if the long-term loss of basal Ca^2+^ level is age dependent, we performed similar experiments in adult animals. Acute Sevo exposure also led to a similar extent of decrease in basal Ca^2+^ level in mature astrocytes (**S8A Fig**) compared with developing ones. However, when we performed Sevo/Control exposure in vivo at P56, we found no significant difference in GCaMP6f basal fluorescent intensity in the two group mice measured 7 d later (P63) (**Fig 3E**). Together, these results suggest mature astrocytes are more resistant in response to the decrease of basal Ca^2+^ level than the immature ones following lengthy general anesthesia.

Spontaneous Ca^2+^ signals were analyzed automatically with post manual inspection by using the computational tool suite Functional AStrocyte Phenotyping (FASP) [28]. Similar to previous studies in mature cortical astrocytes [29], developing cortical astrocytes displayed waves (events area, >10 μm^2^) and microdomain signals (events area, 1.5–10 μm^2^). Consistent with the previous work [26], acute Sevo exposure resulted in decreased frequency and amplitude of Ca^2+^ waves (**S8B Fig**). In addition, Sevo exposure in vivo at P7 also resulted in altered spontaneous Ca^2+^ signals properties measured at P14, including amplitude and half-width (**Fig 3F–3H**). We also assessed evoked Ca^2+^ signals and found that the peak amplitude of mGluR2/3 agonist (1S,2S,5R,6S)-2-Aminobicyclo[3.1.0]hexane-2,6-dicarboxylic acid (LY354740)-evoked Ca^2+^ signals and mGluR1/5 agonist (RS)-3,5-Dihydroxyphenylglycine (DHPG)-evoked Ca^2+^ signals were significantly lower in Sevo group mice, whereas ATP-evoked and noradrenaline (NE)-evoked Ca^2+^ signals were similar between the two groups (**Fig 3I** and **3J**). We further evaluated the expression of metabotropic glutamate receptor 3 (mGluR3) and metabotropic glutamate receptor 5 (mGluR5) in cortical astrocytes at P14 by using quantitative reverse transcription PCR (RT-qPCR) for magnetic cell sorting (MACS)-isolated cortical astrocytes (GLAST^+^ cells) from Control and Sevo group mice (see Materials and methods). We found that both mGluR3 and mGluR5 mRNA levels were significantly lower in Sevo group mice (**Fig 3K**).

Together, this dataset suggests that lengthy Sevo exposure disrupts both acutely and chronically the basal Ca^2+^ levels, spontaneous and neurotransmitter-evoked Ca^2+^ transients in developing cortical astrocytes.

### Down-regulation of Ezrin expression with lengthy Sevo exposure

**Fig 4:**
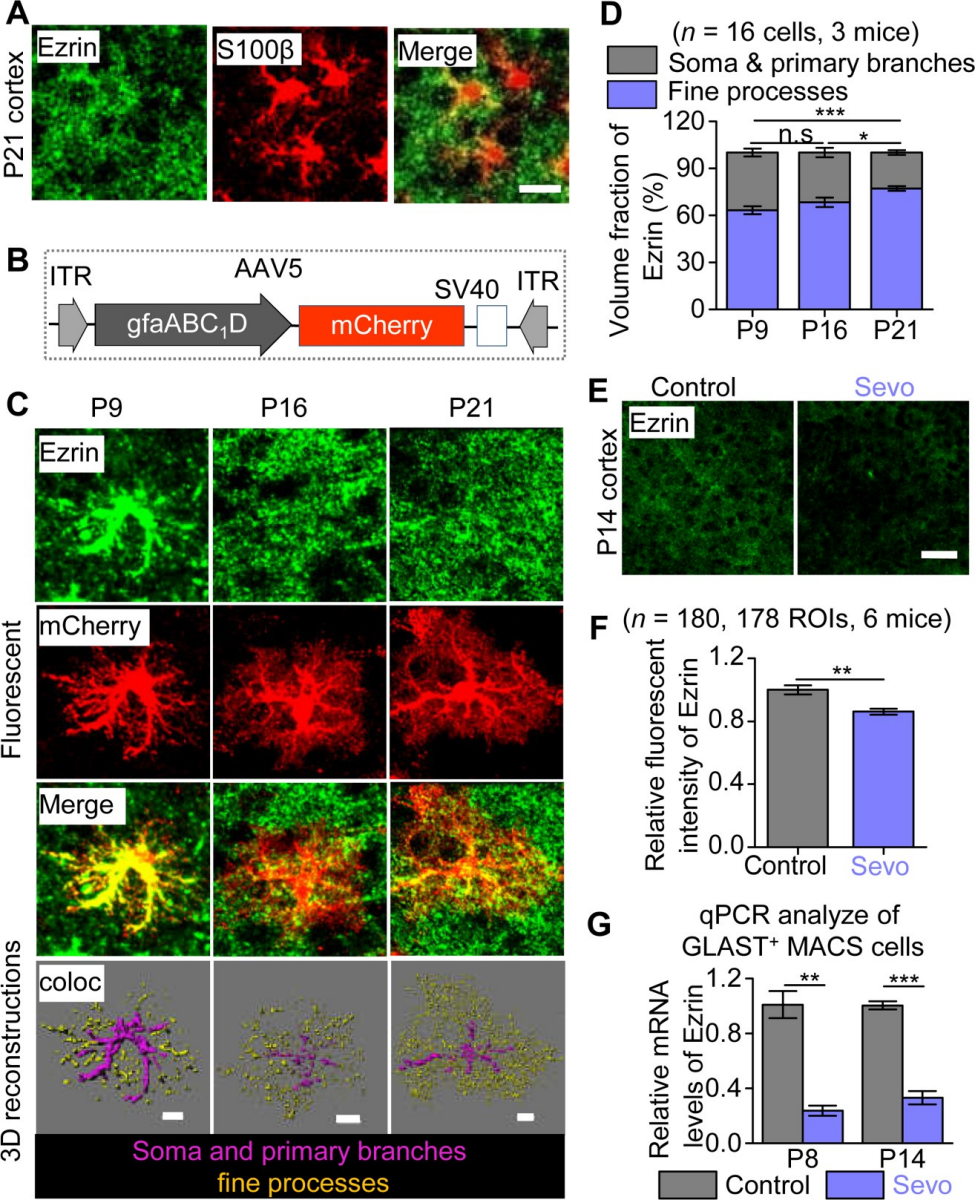
Down-regulation of Ezrin expression with lengthy Sevo exposure. (**A**) Representative images of Ezrin colocalized with S100β. Scale bars, 10 μm. (**B**) Schematic of construction of AAVs. (**C**) Representative fluorescent images of Ezrin- and mCherry-labeled astrocytes, and 3D reconstruction of Ezrin within astrocytic fine processes or soma and primary branches in the somatosensory cortex at P9, P16, and P21. Scale bars, 10 μm. (**D**) Volume fraction of Ezrin within the fine processes, soma, and primary branches of astrocytes (P9 versus P16: *P* = 0.304, P16 versus P21: *P* = 0.04, P9 versus P21: *P* < 0.001, one-way ANOVA followed by post hoc Tukey test). (**E**) Confocal images for Ezrin staining. Scale bars, 50 μm. (**F**) Quantification of Ezrin fluorescent intensity (*P* = 0.007, Mann-Whitney test). (**G**) Quantification of Ezrin mRNA levels of GLAST^+^ MACS cells in the cortex of Control and Sevo group mice at P8 (*n* = 3 independent preparations, *P* = 0.002, unpaired *t* test) and P14 (*n* = 3 independent preparations, *P* = 0.002, unpaired *t* test). **P < 0.01; ***P < 0.001; n.s, not significant. Data are shown as mean ± SEM. Underlying data are available in S1 Data. AAV, adeno-associated virus; coloc, colocalization; GLAST, glutamate-aspartate transporter; ITR, inverted terminal repeat; MACS, magnetic cell sorting; n.s, not significant; qPCR, quantitative PCR; ROI, region of interest; Sevo, sevoflurane.

Ezrin is an actin-binding membrane-bound protein expressed mainly within astrocyte processes in the central nervous system [30] and was found to be required for the structural plasticity of astrocyte processes in culture [31]. Because the loss of morphology in Sevo group mice predominantly occurred in the fine processes (**Fig 1**), we hypothesized that Ezrin may be a key structural determinant of astrocyte fine processes in vivo, and its dysfunction may occur following lengthy general anesthesia. To this end, first, we found that in the somatosensory cortex of P21 mice, Ezrin was only partially colocalized with the astrocyte marker S100β, which labeled mainly the soma and primary branches (**Fig 4A**), whereas most of the Ezrin signals seemed to be peripheral to S100β signals. To precisely quantify the distribution of Ezrin within astrocyte territory, we fluorescently labeled cortical astrocytes by stereotactic injection of AAV5·gfaABC_1_D·mCherry into the cortex of P0 mice and stained Ezrin at P9, P16, and P21 (**Fig 4B and 4C**). We found that the volume fraction of Ezrin was gradually increased in fine processes but decreased in soma and primary branches of cortical astrocytes from P9 to P21 (**Fig 4C and 4D**). At P21, more than 70% of total Ezrin was localized within astrocyte fine processes. Interestingly, the expression of Ezrin in the somatosensory cortex, but not in hippocampal CA1sr or DG, was significantly down-regulated in Sevo group mice compared with Control group mice at P14 (**Fig 4E and 4F; S9 Fig**). Consistent with immunostaining data, the mRNA level of Ezrin in the somatosensory cortex of Sevo group mice at P8 and P14 were also significantly lower, detected by using RT-qPCR for GLAST^+^ MACS cells (**Fig 4G**). Together, our data suggest that Ezrin was developmentally enriched within astrocyte fine processes and was down-regulated in Sevo group mice.

### Astrocyte morphological deficits were induced by down-regulation of Ezrin in a Ca^2+^-dependent manner

**Fig 5:**
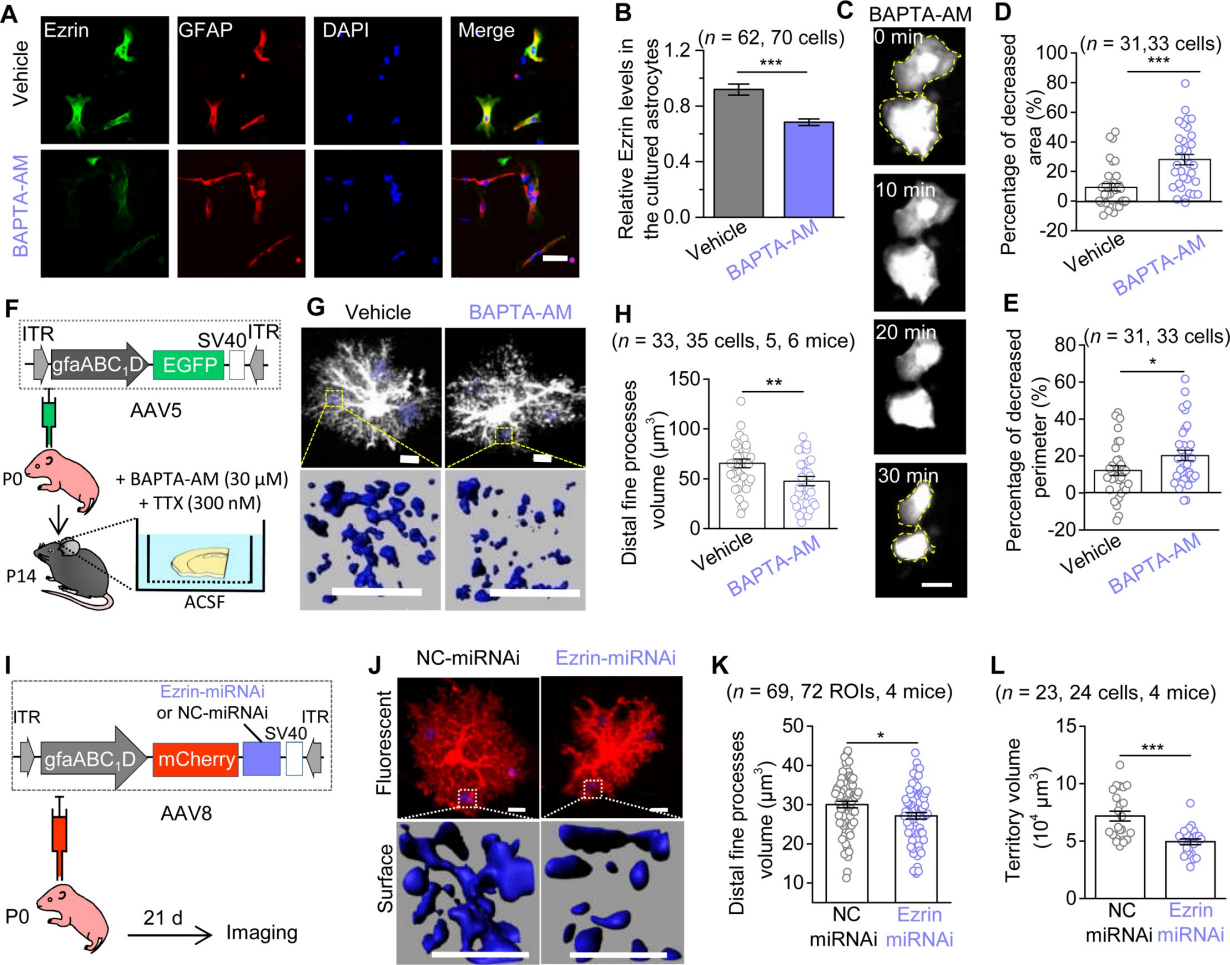
Reducing astrocyte intracellular Ca^2+^ leads to the down-regulation of Ezrin, which produces morphological deficits. (**A**) Representative immunofluorescence images of Ezrin in the primary cultured astrocytes after 1-h incubation with Vehicle (DMSO) or BAPTA-AM (30 μM). Scale bars, 50 μm. (**B**) Quantification of relative fluorescent intensity of Ezrin (*n* = 3 independent preparations; *P* < 0.001, Mann-Whitney test). (**C**) Representative successive imaging of EGFP-labeled astrocytes during BAPTA-AM (30 μM) incubation. Scale bars, 50 μm. (**D, E**) Quantification cultured astrocytes morphology (*n* = 3 independent preparations; **D**: *P* < 0.001, Mann-Whitney test; **E**: *P* = 0.041, unpaired *t* test). (**F**) Diagram illustrates the vector construction, AAV injection in neonate mice, and protocol for brain slices incubation. (**G**) Representative fluorescent images and 3D reconstructed distal fine processes of astrocytes within a 10 by 10 by 10 μm^3^ ROI, 3 ROIs for one cell. Scale bars, 10 μm. (**H**) Quantification of astrocyte distal fine processes (*P* = 0.006, unpaired *t* test). (**I**) The vector construction of AAV8·gfaABC_1_D·mCherry·Ezrin•miRNAi (or NC-miRNAi) and stereotactic injection of AAVs into P0 mice. (**J**) Confocal images and 3D reconstructed astrocytes. (**K**) Quantification of astrocytic distal fine processes volume in NC-miRNAi and Ezrin-miRNAi mice at P21 (*P* = 0.018, unpaired *t* test). Scale bars, 10 μm. (**L**) Quantification of astrocyte territory volume (*P* < 0.001, unpaired *t* test). *P < 0.05, **P < 0.01, ***P < 0.001. Data are shown as mean ± SEM. Underlying data are available in S1 Data. AAV, adeno-associated virus; BAPTA-AM, 1,2-bis-(o-aminophenoxy)-ethane-N,N,N0 N0-tetraacetic acid, tetra-acetoxymethyl ester; EGFP, enhanced green fluorescent protein; GFAP, glial fibrillary acidic protein; ITR, inverted terminal repeat; miRNAi, microRNA-based RNA interference; NC, negative control; ROI, region of interest.

We then asked whether the down-regulation of Ezrin by early Sevo exposure was due to the disrupted astrocyte Ca^2+^ signals (**Fig 3**). Intracellular Ca^2+^ chelation was achieved by incubating cultured astrocytes with a membrane-permeant Ca^2+^ chelator, 1,2-bis-(o-aminophenoxy)-ethane-N,N,N0 N0-tetraacetic acid, tetra-acetoxymethyl ester (BAPTA-AM, 30 μM). The Ezrin expression in the primary cultured astrocytes was significantly decreased after 1-h incubation with BAPTA-AM (**Fig 5A** and **5B**). Interestingly, BAPTA-AM (30 μM, 30 min) incubation induced marked morphological changes, with decreased surface area and perimeter, revealed by successive imaging of enhanced green fluorescent protein (EGFP) plasmid transfected astrocytes in vitro (**Fig 5C–5E**). Similar morphological deficits were also observed in astrocytes in cortical slices treated with BAPTA-AM, in which AAV5·gfaABC_1_D·EGFP was microinjected into the mouse cortex at P0 to sparsely label astrocytes in vivo (**Fig 5F**). Astrocytes’ fine process volume was decreased after BAPTA-AM incubation for 1 h in the presence of TTX (**Fig 5G** and **5H**). Thus, our data so far suggest that reducing astrocyte intracellular Ca^2+^ was sufficient to produce the loss of Ezrin and the loss of astrocyte morphology.

Did the loss of Ezrin lead to the loss of astrocyte morphology? We sought to genetically knock down Ezrin within cortical astrocytes in vivo using microRNA-based RNA interference (miRNAi) delivered by adeno-associated virus (AAV). We designed AAV8·gfaABC_1_D·mCherry·Ezrin-miRNAi (Ezrin-miRNAi) and its negative control AAV8·gfaABC_1_D·mCherry·NC-miRNAi (NC-miRNAi) (see Materials and methods), and performed microinjections into the cortex of P0 mice (**Fig 5I**). Ezrin-miRNAi injection resulted in a significant down-regulation of Ezrin expression (by approximately 26%) in the somatosensory cortex of mice at P21, assessed by Ezrin immunostaining (**S10A** and **S10B Fig**). The miRNAi expression is highly specific to astrocytes, as 94.4% of mCherry^+^ cells colocalized with S100β and only 5.6% of which colocalized with neuronal nuclei (NeuN) (**S10C** and **S10D Fig**). Furthermore, Ezrin-miRNAi or NC-miRNAi injection into the cortex of P0 mice did not cause apparent astrogliosis at P21 (**S10E Fig**). As expected, Ezrin knock-down (KD) resulted in decreased astrocytes fine processes and territory volume in P21 mice, which can be easily detected by morphological reconstruction based on mCherry fluorescence (**Fig 5J–5L**), whereas the soma volume was slightly increased and primary branches volume and number were unchanged (**S10F** and **S10G Fig**).

Together, our data suggest that suppressing intracellular Ca^2+^ signals in astrocytes produced severe astrocyte morphological deficits along with the down-regulation of Ezrin, and importantly, KD of Ezrin in vivo was sufficient to produce the loss of astrocyte fine processes, which was very similar to the structural loss of the cortical astrocytes from Sevo group mice.

### Overexpression of Ezrin within astrocytes rescued astrocytic and neuronal dysfunctions and fully corrected social behavior deficits in Sevo group mice

**Fig 6:**
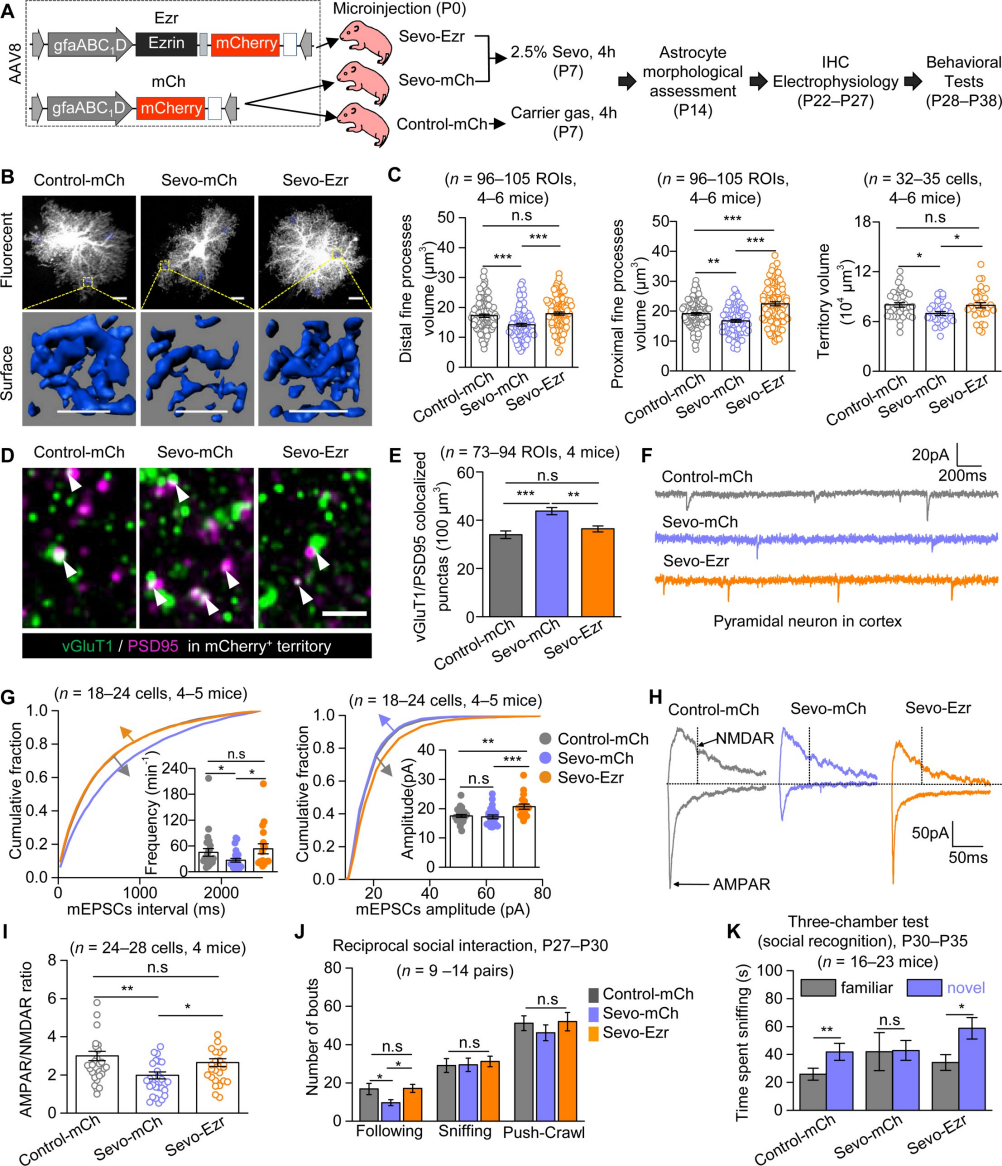
Ezrin overexpression was sufficient to correct cellular and behavioral deficits induced by lengthy Sevo exposure. (**A**) The virus constructions of Ezr and mCh, animal grouping, and the experimental protocols. (**B**) Representative confocal images and 3D reconstructed distal fine processes of sparsely labeled cortical astrocytes at P14. Scale bars, 10 μm. (**C**) **Left**, quantification for experiments in **B**. (**Left**: Control-mCh versus Sevo-mCh: *P* < 0.001; Sevo-mCh versus Sevo-Ezr: *P* < 0.001; Control-mCh versus Sevo-Ezr: *P* = 0.303; Kruskal-Wallis test followed by post hoc multiple comparison test. **Middle:** Control-mCh versus Sevo-mCh: *P* = 0.005; Sevo-mCh versus Sevo-Ezr: *P* = 0; Control-mCh versus Sevo-Ezr: *P* < 0.001; one way ANOVA followed by post hoc Tukey test. **Right:** Control-mCh versus Sevo-mCh: *P* = 0.034; Sevo-mCh versus Sevo-Ezr: *P* = 0.049; Control-mCh versus Sevo-Ezr: *P* = 0.990; one-way ANOVA followed by post hoc Tukey test). (**D**) Confocal images of vGluT1 (green) and PSD95 (magenta) within mCherry-positive (mCherry^+^) territories in the three groups at P22–P27; the white arrows showed the vGluT1/PSD95 colocalized puncta. Scale bar, 2 μm. (**E**) Quantification for experiments in **D** (Control-mCh versus Sevo-mCh: *P* < 0.001; Sevo-mCh versus Sevo-Ezr: *P* = 0.001; Control-mCh versus Sevo-Ezr: *P* = 0.183; Kruskal-Wallis test followed by post hoc multiple comparison test). (**F**) Representative mEPSCs traces of L3-5 cortical pyramidal neurons from the three groups. (**G**) **Left**, cumulative distributions and average (Control-mCh versus Sevo-mCh: *P* = 0.011; Sevo-mCh versus Sevo-Ezr: *P* = 0.013; Control-mCh versus Sevo-Ezr: *P* = 0.891; Kruskal-Wallis test followed by post hoc multiple comparison test) of mEPSCs frequency. The traces of cumulative distribution of mEPSCs intervals in Control-mCh and Sevo-Ezr groups were overlapped. **Right**, cumulative distributions and average (Control-mCh versus Sevo-mCh: *P* = 0.492; Sevo-mCh versus Sevo-Ezr: *P* < 0.001; Control-mCh versus Sevo-Ezr: *P* = 0.005; Kruskal-Wallis test followed by post hoc multiple comparison test) of mEPSCs amplitude. The traces of cumulative distribution of mEPSCs amplitude in Control-mCh and Sevo-mCh groups were overlapped. (**H**) Representative eEPSCs traces of L3-5 cortical pyramidal neurons from the three groups at P22–P27. (**I**) Quantification of AMPAR/NMDAR ratio (Control-mCh versus Sevo-mCh: *P* = 0.002; Sevo-mCh versus Sevo-Ezr: *P* = 0.018; Control-mCh versus Sevo-Ezr: *P* = 0.490; Kruskal-Wallis test followed by post hoc multiple comparison test). (**J**) Quantification for reciprocal social interaction test (Control-mCh versus Sevo-mCh: *P* = 0.039; Sevo-mCh versus Sevo-Ezr: *P* = 0.020; Control-mCh versus Sevo-Ezr: *P* = 0.910; Kruskal-Wallis test followed by post hoc multiple comparison test). (**K**) Quantification for social recognition in the three-chamber test (Control-mCh: *P* = 0.002, paired *t* test; Sevo-mCh: *P* = 0.206, Mann-Whitney test; Sevo-Ezr: *P* = 0.018, Mann-Whitney test). *P < 0.05, **P < 0.01, ***P < 0.001. Data are shown as mean ± SEM. Underlying data are available in S1 Data. AAV, adeno-associated virus; AMPAR, α-amino-3-hydroxy-5-methyl-4-isoxazole propionate receptor; eEPSC, evoked excitatory postsynaptic current; Ezr, AAV8•gfaABC_1_D•Ezrin•P2A•mCherry; IHC, immunohistochemistry; L3-5, layer 3–5; mCh, AAV8•gfaABC_1_D•mCherry; mEPSC, miniature excitatory postsynaptic current; NMDAR, N-methyl-D-aspartic acid receptor; ROI, region of interest; PSD95, postsynaptic density protein 95; Sevo, sevoflurane; vGluT1, vesicular glutamate transporter 1.

In the last set of experiments, we designed AAV8·gfaABC_1_D·Ezrin·P2A·mCherry (Ezr) to specifically enhance Ezrin expression in cortical astrocytes and used AAV8·gfaABC_1_D·mCherry (mCh) as the control virus (**Fig 6A**). In mice with Ezr or mCh microinjection into the cortex at P0, we detected wide and robust viral expression (indicated by mCherry) in the cortex in both groups of mice from P14 up to P40 (**S11A Fig**). Ezr mice exhibited marked increase of Ezrin expression measured at P14 (**S11B** and **S11C Fig**). We also confirmed that the Ezr expression mediated by AAV injection was specific to astrocytes (**S11D** and **S11E Fig**). Thereafter, we tested if the overexpression of Ezrin could rescue the cellular and behavioral deficits in mice with lengthy Sevo exposure (**Fig 6**). In the subsequent study, mice were divided into three groups: mice with mCh injection at P0 and with carrier gas exposure at P7 (Control-mCh mice), mice with mCh injection at P0 and with Sevo exposure at P7 (Sevo-mCh mice), and mice with Ezr injection at P0 and with Sevo exposure at P7 (Sevo-Ezr mice) (**Fig 6A**). First, we analyzed the morphology of mCherry-positive astrocytes, i.e., cells targeted by the viruses, in the three groups of mice using lucifer yellow iontophoresis at P14 (**S12A Fig**). Consistent with the data presented in **Fig 1**, we detected a significant decrease in fine processes and cellular territory volume in Sevo-mCh mice compared with Control-mCh mice (**Fig 6B** and **6C**). Remarkably, this decrease was significantly ameliorated in Sevo-Ezr mice (**Fig 6B** and **6C**). No differences in soma and main branches were detected across groups (**S12B–S12E Fig**). Next, we quantified the synaptic density by the colocalization of presynaptic marker vesicular glutamate transporter 1 (vGluT1) and postsynaptic marker postsynaptic density protein 95 (PSD95). We analyzed the synaptic density both in the territories of virus-expressing astrocytes (which are mCherry^+^) and in the territories without mCherry expression (mCherry^−^). Within the mCherry^+^ area, we found that the density of excitatory synapses was significantly higher in Sevo-mCh mice compared with those in Contro-mCh mice, and this abnormal increase was corrected by Ezrin overexpression in Sevo-Ezr mice (**Fig 6D** and **6E**). In the mCherry^−^ territories, however, aberrant increase of excitatory synaptic density was observed in Sevo-exposed mice (Sevo-mCh and Sevo-Ezr mice), as compared with carrier gas exposed mice (Control-mCh mice) (**S13A–S13D Fig**). Reduced mEPSCs frequency and AMPAR/NMDAR ratio were found in Sevo-mCh mice compared with Control-mCh mice, and this abnormal decrease was corrected in Sevo-Ezr mice (**Fig 6F–6I**). Ezrin overexpression also induced increase in mEPSCs amplitude (**Fig 6G Right**) and AMPAR-eEPSCs decay (**S13F Fig**). Unchanged NMDAR-eEPSCs decay and input resistance were found across the three groups (**S13E–S13G Fig**). Last, social behaviors were assessed in the three groups of mice. In the reciprocal social interaction test, significantly fewer following behaviors were identified in Sevo-mCh mice as compared with Control-mCh mice, and this abnormality was fully rescued in Sevo-Ezr mice (**Fig 6J**). In the three-chamber test, impaired social recognition in Sevo-mCh mice was observed, which was fully corrected in Sevo-Ezr mice (**Fig 6K**). No abnormal sociability was observed across groups (**S13H Fig**). The virus expression of all the mice involved in the behavioral tests was confirmed at P40. Taken together, the data above show that the astrocytic Ezrin overexpression was sufficient to restore astrocytic and neuronal structure and function and fully corrected the social behavioral deficits in mice with neonatal lengthy Sevo exposure.

## Discussion

**Fig 7:**
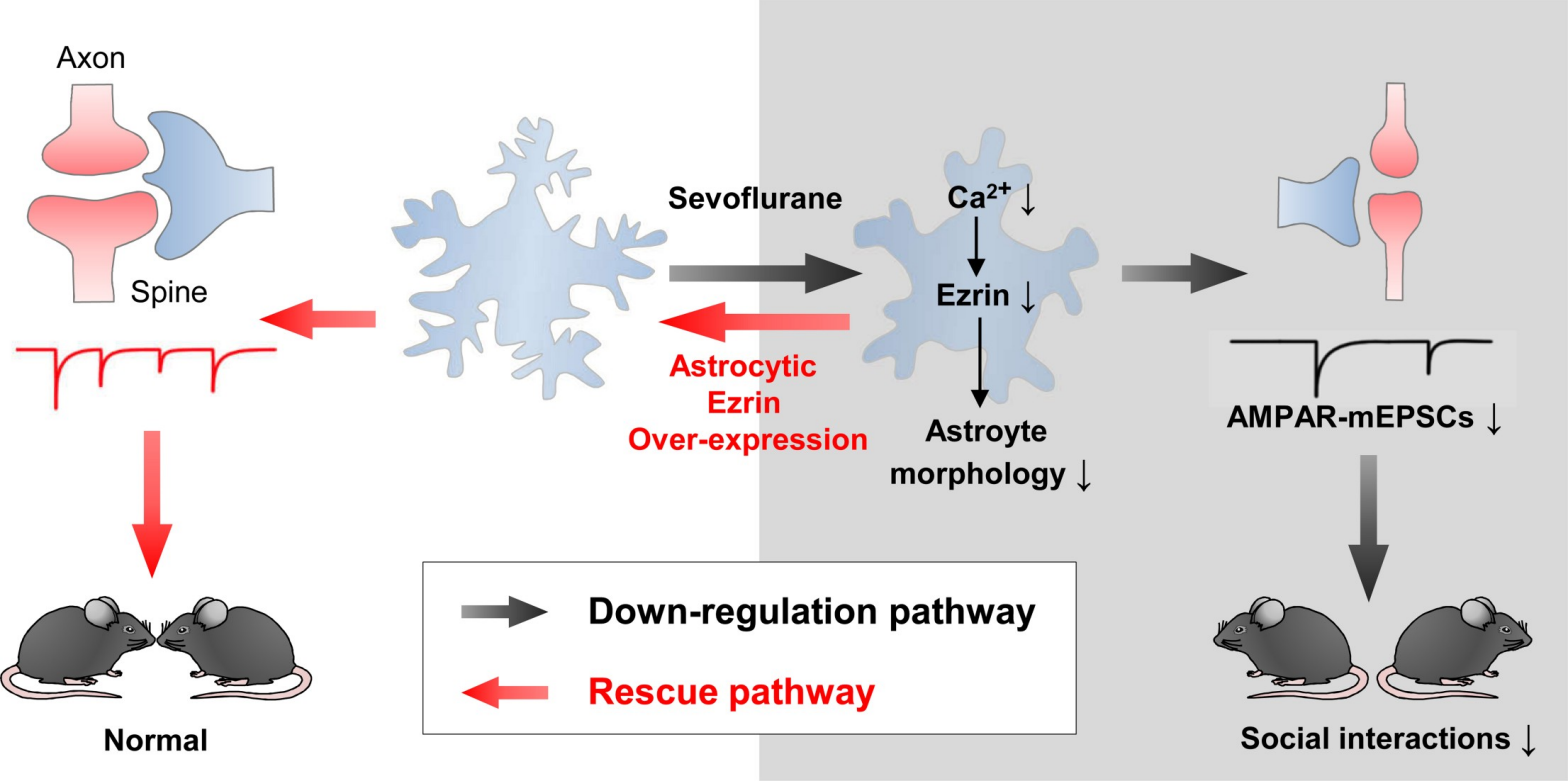
Summary for the key findings in the current study. Lengthy Sevo exposure disrupted astrocyte intracellular Ca^2+^ signals, which led to the down-regulation of Ezrin. The loss of Ezrin resulted in astrocyte morphological deficits, producing aberrant synaptic structure and function, which contributed to the deficits in social behaviors. Overexpression of astrocyte Ezrin not only rescued astrocytic and neuronal dysfunctions but also fully corrected deficits in social behaviors in Sevo group mice. AMPAR, α-amino-3-hydroxy-5-methyl-4-isoxazole propionate receptor; mEPSCs, miniature EPSCs; Sevo, sevoflurane.

There are several key findings from this study, some of which are schematized in **Fig 7.** First, we reported in detail how lengthy Sevo exposure disrupted astrocyte morphogenesis. Compromised astrocyte morphogenesis was associated only with a relatively long exposure (4 h) and occurred only in the developing brain, not in the mature brain. Second, we showed that the Sevo-induced aberrant synaptogenesis and astrocytic morphological deficits were spatiotemporally correlated. Third, we showed Sevo group mice exhibited social behavioral deficits. Fourth, we revealed the underlying mechanisms for compromised astrocyte morphogenesis, in which Sevo targeted astrocyte Ca^2+^ signaling, and hereby disrupted Ezrin expression, which functioned as a key regulator of astrocyte morphogenesis in vivo. We further showed Ezrin was developmentally up-regulated in astrocyte fine processes, and the disruption of which predominantly resulted in the loss of the fine processes. Fifth, we showed that overexpression of astrocyte Ezrin rescued astrocytic and neuronal dysfunctions and fully corrected deficits in social behaviors in Sevo group mice.

Apoptosis and abnormal neural circuit formation, including aberrant neurite growth, guidance, and synaptic connections, are the major molecular and cellular phenotypes reported in the GAs-induced neurotoxicity [1]. In the current study, we focused on the structural and functional deficits in astrocytes and neurons that are highly relevant to neural circuit formation and functions. The initial goal was to evaluate how lengthy Sevo exposure induces astrogliosis. To our surprise, in the Sevo group mice, cortical astrocytes displayed loss of fine processes without apparent astrogliosis. The loss or delayed growth of astrocyte fine processes, although subtle, was identified using two independent methods: single astrocyte fluorescent labeling and SBF-SEM. At P21, structural loss in astrocytes was not visible under light microscopy but remained profound under SBF-SEM. Our study indicates that the detailed morphological analysis of astrocytes in addition to conventional staining for astrogliosis or cytoskeletal impairment is needed for a better understanding of astrocyte biology in the context of diseases or injuries.

Astrocyte morphological deficits displayed exposure-length, age, and brain-region dependence. The exposure-length and age dependence are consistent with the clinical findings so far, including the MASK study, in which only infants or children with repeated or prolonged general anesthesia are associated with aberrant neurological behaviors [6,32]. Consistent with a previous animal study with propofol [33], we found Sevo lengthy anesthesia induced an increase of dendritic spines and synapses at P21 in the cortex, but decreases were also reported [34,35]. We did not observe significant astrocyte and neuron structural deficits in developing hippocampal CA1sr and DG-mo regions, nor hippocampus-associated cognitive deficits, whereas a few studies found abnormal synaptic growth [18], altered synaptic plasticity [34] in the hippocampus, as well as hippocampus-associated cognitive deficits [18,36]. The inconsistency may be due to different anesthetic agents (isoflurane, Sevo, propofol, etc.) used, different exposure conditions (different concentration and exposure time, repeated exposure, or single exposure) or different types of cells examined (pyramidal neurons or granule cells), or any other different experimental settings. Regardless, with experiments performed with intact tissue preparation or in vivo under our experimental paradigms, we revealed both neuronal and astrocytic deficits that were highly correlated in the somatosensory cortex and likely in other cortical areas. Furthermore, our behavioral data highly suggest that, in additional to cognitive functions, the social behaviors of infants/children with multiple/lengthy general anesthesia should also be evaluated in depth in clinical trials.

Astrocytes use intracellular Ca^2+^ signals as one of their primary forms to engage in neural circuits, and the disruption of which alters behavior [37] and contributes to diseases and injuries [38]. We found that Sevo disrupted astrocyte intracellular Ca^2+^ transients and basal Ca^2+^ level both acutely and chronically. Interestingly, most of the clinically used GAs suppress Ca^2+^ transients in vivo [26]; therefore, it is likely that astrocytes Ca^2+^ may represent a common pathway for GAs-induced neurotoxicity. Further experiments are needed to test if other GAs with distinct action mechanisms, including isoflurane, propofol, and ketamine, disrupt Ca^2+^ homeostasis in developing astrocytes. The decrease of basal Ca^2+^ level by Sevo is of particular interest, because it has been recently shown that basal Ca^2+^ level decreased from P15 to P21 in mouse hippocampus, suggesting that there is an intrinsic regulatory mechanism for basal Ca^2+^ level during development [39]. The molecular pathway(s) by which Sevo disrupts astrocyte Ca^2+^ efflux/influx are currently unknown. A wide range of ion channels and transporters have known sensitivities to inhaled anesthetics [40], some of which are expressed in astrocytes, such as sodium–calcium exchangers (NCX), nicotinic receptors, background two-pore domain K^+^ channels, etc. The net effects of Sevo on astrocyte Ca^2+^ likely involve multiple targets and multiple mechanisms, which require further studies to clarify.

Actin-binding proteins of the Ezrin, Radixin, and Moesin (ERM) family play key roles in the molecular mechanisms of cell motility, process formation, and tumorigenesis. In the central nervous system, Ezrin was found specifically localized to fine astrocytic processes [30,31]. We extended the previous findings by showing that the development profile of Ezrin expression was correlated with astrocyte morphological maturation, a process involved in a substantial growth of the fine processes, and we provided direct evidence for the functional involvement of Ezrin in astrocyte fine structure growth by showing that the disruption of Ezrin in vivo predominantly affected the formation of the fine processes. Astrocyte fine processes display actin-dependent structural plasticity that is regulated by synaptic activity through astrocyte metabotropic glutamate receptors and intracellular Ca^2+^ signaling, and the astrocyte structural plasticity in turn controls synapse stability [41]. Therefore, in subsequent studies, it would be of particular interest to test if Ezrin is involved in neural activity–dependent structural plasticity at the synaptic scale, thereby participating in astrocyte-neuron dynamic interactions in vivo and, ultimately, in behavior.

Lengthy Sevo exposure produced an intriguing and apparently paradoxical neuronal phenotype: an increase of total synapses with reduced AMPAR-mediated synaptic function. The most straightforward explanation is that there was a much greater proportion of silent synapses in Sevo group mice, whereas the proportion of functional ones was reduced. Here, we focus our discussion on astrocytic roles in forming functional and nonfunctional synapses. In the developing brain, newly formed glutamatergic synapses are often associated with silent postsynaptic spines that lack functional AMPAR-mediated transmission. Most of these silent synapses are eliminated, but some are specifically selected for AMPAR un-silencing and lead to stabilization during development [42]. Increased (less functional) synapses with reduced functions likely resulted from an imbalance among all three key processes required for proper synaptogenesis: synaptic formation, stabilization, and elimination. There are a few astrocyte-based mechanisms for synaptogenesis. For instance, astrocyte-secreted molecules, including thrombospondins and hevin, promote structurally normal but functional silent synapses, while glypican 4 and glypican 6 convert silent synapses into functional ones by increasing the surface level of AMPAR, and multiple epidermal growth factor-like domains 10 (MEGF10) and c-Mer tyrosine kinase receptor (MERTK) mediate synapse elimination through phagocytosis [43]. All the signaling pathways above require proper spatial interactions between neurons and astrocytes. We hypothesize that astrocyte structural deficits resulted in an increase or decrease of astrocyte–neuron signaling efficiency due to the altered spatial interactions between astrocytes and neurons at the synaptic level. In support, recent work by Stogsdill and colleagues [44] demonstrated that astrocyte–neuron adhesions control astrocyte morphogenesis and thereby control synaptogenesis.

Unexpectedly, our data uncover that, in addition to neurons, astrocytes may represent important targets for GAs to exert toxic effects. Postoperative cognitive dysfunction (POCD) is a chronic decline in cognitive function also associated with anesthesia/surgery. It was found that POCD was associated with astrocytic decreased Ca^2+^ signaling along with enhanced synaptic transmission in the mouse hippocampus [45]. The causal relationship between astrocyte and neuron dysfunctions in POCD remains to be established. More broadly, in addition to the earlier studies in Huntington disease (HD) mouse models in which astrocytes displayed functional [46] and morphological [21] deficits before astrogliosis, our study presented here reinforces the idea that astrocyte fine structural integrity may represent a therapeutic target in a variety of neurological and psychiatric diseases.

## Materials and methods

### Ethics statement

All experiments with mice were approved by the Animal Research Committee at the West China Hospital of Sichuan University (protocol 2018159A). For tissue collection, mice were given a lethal dose of pentobarbital intraperitoneally.

### Animals

C57BL/6 mice were housed in a temperature- and humidity-controlled room with a 12-h light–dark cycle and provided with ad libitum access to water and food. Both sexes were equally presented in all experiments.

### Sevo exposure protocol

P7 mouse littermates were randomly assigned to two groups. In Sevo group, mice were exposed to 2.5% Sevo (HENGRUI MEDICINE, Lianyungang, China) carried in 30% O_2_/70% N_2_ at a flow rate of 1.5 L/min for 4 h. An agent-specific vaporizer (Vanbon, ibis 200) was used to deliver Sevo. Gas was monitored by an agent gas monitor (Philips). The temperature of the anesthesia chamber was maintained at 36–37°C by using a heating pad. Mice were continually monitored and recorded for skin temperature and respiratory rate during anesthesia. Animals were returned to their mothers upon regaining righting reflex. Intracardiac puncture was used to collect left ventricular blood samples for arterial blood gas analysis (Mindray, BC-3000).

### Intracellular Lucifer yellow dye filling and morphological 3D reconstructions

#### Astrocytes

Intracellular dye filling was performed on the lightly fixed brain slices from the L3-5 of primary somatosensory cortex, hippocampal CA1sr, and hippocampal DG of mice at P8, P14, and P21 using the protocols as previously reported. In the Ezrin overexpression experiments, mCherry-positive astrocytes were also labeled with Lucifer yellow, because the autofluorescence of mCherry was insufficient to map the fine processes of astrocytes at P14 (**S12A Fig**). After injection, the confocal imaging stacks were collected with a Z-step size of 0.25 μm under a multiphoton microscope (Nikon A1R^+^). Three-dimensional reconstructions were processed offline using Imaris 7.4.2 (Bitplane, South Windsor, CT) as reported previously [21]. To quantify the fine processes of astrocytes, six randomly chosen 5 by 5 by 5 μm ROIs devoid of the soma and large branches were reconstructed with the Local Contrast method. Three of these ROIs that near the soma were termed as proximal fine processes, and the other three ROIs that located in the edge of astrocyte were termed as distal fine processes. Three-dimensional surface rendering of astrocytes for calculating the territory volume was achieved similarly, utilizing Background Subtraction of Imaris.

#### Dendritic spines

Dendritic spines visualization using intracellular Lucifer yellow dye filling was performed similarly as previously described [47]. Consecutive stacks of images with Z-steps of 0.25 μm were acquired at high magnification (60× glycerol, 5× optical zoom) using a multiphoton microscope (Nikon A1R^+^). The image stacks were then reconstructed and analyzed by using the Filament function of Imaris. Dendritic spine density was determined by counting the number of dendritic spines per 10 μm of dendritic length, starting from 50 μm to 120 μm away from the soma, where the dendritic spine density was reported with low variety. For apical dendrites, the 70-μm-long segment was also analyzed every 10 μm of dendritic length. For spine classification, the Filament function of Imaris was used; stubby: length (spine) < 1.5 and max width (head) < mean width (neck) × 1.2; mushroom: max width (head) > mean width (neck) × 1.2 and max width (head) > 0.3; the rest were defined as long-thin [18]. We only quantified the total and mushroom spine density, but the stubby and long-thin were difficult to classify in many cases due to the limited image resolution.

### SBF-SEM

Mice were euthanized with 100 mg/kg pentobarbital and transcardially perfused with 40 mL of fixative solution (2% paraformaldehyde Sigma-Aldrich #158127) and 2.5% glutaraldehyde (Sigma-Aldrich #G7651) in a 0.1 M phosphate buffer (PB), pH 7.4. Brains were sliced into 100-μm coronal sections and we then further dissected the L3-5 of primary somatosensory cortex. Tissues were postfixed in fixative solution for 48 h post euthanasia at 4°C. After washing with PB (pH 7.4) three times, tissues were fixed with osmium-ferricyanide followed by thiocarbohydrazide treatment, and then further fixed with 1% aqueous osmium tetroxide. Samples were then incubated overnight in 2% aqueous uranyl acetate at 4°C. Next, samples were dehydrated through a series of alcohol solutions and embedded in SPI-Pon 812-substitute resin. The block was carefully trimmed for focused ion beam scanning electron microscopy imaging with Helios NanoLab 600i (FEI). Samples were imaged at accelerated voltage 4 keV, beam current 0.34 nA, at 3.37 nm/pixel resolution, with a horizontal field width of 13.8 μm and slice thickness of 60 nm [48]. Each image series contains 90–120 sections with a total volume of 3,313.3–3,542.2 μm^3^ in Sevo and Control groups at P14 and P21. Image series were registered and then analyzed using Reconstruct software version 1.1 (https://synapseweb.clm.utexas.edu/software-0). Astrocytic profiles are clearly identifiable in serial images owing to their pale cytoplasm, irregular contouring border, and the absence of vesicles as well as synapses. Only asymmetric synapses identified by the shape of their axonal and dendritic spines and their synaptic vesicles were included in the quantification. We randomly selected fields of 16 μm^2^ and thickness of 1.8 μm (volume 28.8 μm^3^) or fields of 25.1 μm^2^ and thickness of 3 μm (volume 75.3 μm^3^) from blocks in Sevo and Control group mice for reconstruction. These volumes did not include any large dendritic profiles or soma of neurons, glia, or endothelial cells. To quantify astrocyte–synaptic cleft contacts, only astrocytic process endings visually directly apposed PSDs, i.e., with no intervening elements of the neuropil, were counted. Cross-sectioned astrocyte perimeters of the synaptic cleft were computed by assigning the contact point between the astrocytic membrane and the synaptic cleft in individual sections, then summing over all sections. The mushroom dendritic spines were recognized as the presence of a spine apparatus and a complex PSD (perforated, U-shaped, or segmented), whereas other spines had only macular PSDs and no spine apparatus [49]. The stubby spines were not separately classified from long-thin spines.

### AAVs generation

The pZac2.1.gfaABC_1_D·cyto-GCaMP6f plasmid was a gift from Dr. Baljit Khakh (UCLA) and was sent to Dr. Biao Dong’s Lab (Sichuan University) for AAV packaging. The AAV5·gfaABC_1_D·EGFP and AAV5·gfaABC_1_D·mCherry were gifts from Taitool Bioscience. To achieve astrocyte-specific Ezrin KD using microRNA-based silencing technique, we used the BLOCK-iT Pol II miR RNAi Expression Vector Kits (Invitrogen). Six pre-miRNA sequences for Ezrin (Ezrin-miRNA) and negative control sequence (NC-miRNA) were designed (Invitrogen’s RNAi Designer), synthesized, and cloned into pAAV·CMV_bGI·mCherry·miRNAi vector (Taitool Bioscience). The KD efficiency was then evaluated by cotransfecting EGFP-tagged Ezrin with the Ezrin miRNA vectors separately in human embryonic kidney (HEK293) cells, and the KD efficiency was indicated by the reduction of the fluorescence signal expressed by the EGFP-Ezrin vector. The most effective sequence was chosen as follows: Ezrin-miRNA, 5′-AAAGTCAGGTGCCTTCTTGTC-3′, and NC-miRNA, 5′-AAATGTACTGCGCGTGGAGAC-3′. The selected oligos were then cloned into the linearized pAAV·gfaABC_1_D·mCherry·miRNAi vector (Taitool Bioscience) using T4 DNA ligase. For overexpression of astrocytic Ezrin, the CDS of mouse Ezrin gene (Ezr, NCBI gene ID: 22350) was synthetized and cloned into pAAV·gfaABC_1_D·Ezrin·P2A·mCherry (short for Ezr), where Ezrin and mCherry was link by P2A. The control vector was pAAV·gfaABC_1_D·mCherry (mCh). The plasmids were packaged into the AAV8 virus by calcium phosphate transfection with capsid and helper vectors on HEK293 cells. The collected viruses were purified by iodixanol density gradient centrifugation. The titer of AAV8·gfaABC_1_D·mCherry·Ezrin-miRNAi, AAV8·gfaABC_1_D·mCherry·NC-miRNAi, AAV8·gfaABC_1_D·Ezrin·P2A·mCherry, and AAV8·gfaABC_1_D·mCherry was determined by qPCR.

### AAVs microinjections

P0 neonates were under cryo-anesthesia before injection as previously reported [50]. Viral injections were performed by using a stereotaxic apparatus (RWD, China) to guide the placement of a Hamilton syringe fixed with beveled glass pipettes (Sutter Instrument, 1.0-mm outer diameter) into the cortex. The injection site was located at half of the distance along a line defined between each eye and the lambda intersection of the skull. The needle was held perpendicular to the skull surface during insertion to a depth of approximately 0.2 mm. A total of 1.0 μL of AAV5·gfaABC_1_D·cyto-GCaMP6f (GCaMP6f) (2.0 × 10^13^ gc/mL), 1.0 μL of AAV5·gfaABC_1_D·mCherry (3.46 × 10^12^ gc/mL), 1.0 μL of AAV5·gfaABC_1_D·EGFP (2 × 10^13^ gc/mL), 0.6 μL of Ezrin-miRNAi (5.5 × 10^12^ gc/mL), 0.6 μL of NC-miRNAi (5.4 × 10^12^ gc/mL), 1.0 μL AAV8·gfaABC_1_D·Ezrin·P2A·mCherry (4.62 × 10^12^ gc/mL), or 0.6 μL AAV8·gfaABC_1_D·mCherry (4.0 × 10^12^ gc/mL) was slowly injected into both sides of the hemisphere. Glass pipettes were left in place for at least 5 min. After injection, pups were allowed to completely recover on a warming blanket and then returned to the home cage.

### Acute brain slice preparation and electrophysiology

Acute slices were prepared as previously described [51]. L3-5 pyramidal neurons in somatosensory cortex in slices were visualized with infrared optics on an upright microscope (BX51WI, Olympus). Clampex 10.4 software and a MultiClamp 700B amplifier (Molecular Devices) were used for electrophysiology (Molecular Devices). All recordings were carried out at room temperature. Recordings were filtered at 2 kHz, digitized at 10 kHz, and acquired with Digidata 1440A (Molecular Devices). The recording pipettes (3–4 MΩ) were pulled from borosilicate glass by using a Flaming-Brown horizontal puller (Model P97, Sutter Instruments). For mEPSCs recordings, the intracellular solution in the patch pipette contained the following (in mM): 130 KCl, 2 NaCl, 10 HEPES, 5 EGTA, 2 Mg-ATP, 0.5 CaCl_2_, pH 7.3, adjusted with KOH. For eEPSCs recordings, the intracellular solution in the patch pipette contained the following (in mM): 120 cesium methanesulfonate, 15 CsCl, 8 NaCl, 10 HEPES, 0.2 EGTA, 10 TEA-Cl, 2 Mg-ATP, 0.3 Na_2_-GTP, 3 QX-314, pH 7.3, adjusted with CsOH. The initial access resistances were <20 MΩ for all cells; if this changed by >25%, the cell was discarded. Synaptic currents were collected for 3–5 min for each cell. Analysis was carried out by using Mini Analysis Program (version 6.0.3; Synaptosoft, Leonia, NJ). Only events greater than 8 pA, with a rise time less than 2 ms and decay time less than 10 ms, were included in the analysis. For eEPSCs, a bipolar stimulating electrode was placed in the somatosensory cortex. The recording pipettes were typically located 250–300 μm away from the stimulation site. Only single-peaked responses were included for analysis. The time constant for the decay of eEPSCs was determined by fitting the decay to a single exponential by using pCLAMP10.7 software.

### Ca^2+^ imaging

Acute brain slices from mice injected with GCaMP6f were prepared for Ca^2+^ imaging. Slices were imaged using a Nikon A1R^+^ multiphoton microscope with 40× (NA 0.8) water immersion objective (Nikon), using the 488-nm laser. Slices were continuously superfused at 1–2 mL/min with oxygenated aCSF at room temperature. Images were typically 512 × 512 pixels/frame with 1–2× optical zoom at a scan rate of 1 frame/s. Drugs (LY354740: 10 μM, Tocris; DHPG: 100 μM, Tocris; ATP: 300 nM, Sigma-Aldrich; NE: 20 μM, Abisin) were loaded by bath application. For acute administration of Sevo, 2.5% Sevo was equilibrated in bathing solutions in a reservoir by passing air (flow rate, 0.5 L/min) through a calibrated vaporizer for at least 30 min before entering a recording chamber. Samples of the superfusate in the recording chamber were collected for the measurement of the Sevo concentration by gas chromatography. The mean Sevo concentration was 0.36 ± 0.03 mM. Recordings were excluded from analysis if the cell drifted out of frame or out of the z-plane. Lateral drifts in astrocyte position were corrected with the TurboReg Plugin in ImageJ. Spontaneous Ca^2+^ signals were analyzed by MATLAB (R2015a, MathWorks) using custom-written scripts called “Functional Astrocyte Phenotyping (FASP)” [28]. A signal was declared as a Ca^2+^ transient if it exceeded the detection by greater than twice of the baseline noise (SD).

### Tissue dissociation, astrocyte sorting, and qPCR

The cortical hemispheres from Control and Sevo group mice at P14 were dissociated following published guidelines [52] with slight modifications. Briefly, the cortex from four mice were dissected and digested together for 45 min at 37°C with 10 mL of enzyme solution (Dulbecco’s minimum essential medium [DMEM], 50 mM EDTA, 50 U/mL DNase-1, 0.1% collagenase, and 0.05% trypsin) while bubbling with 5% CO_2_/95% O_2_. After digestion, the tissue was mechanically dissociated and filtered with a 70-μm mesh. Astrocytes were separated by a MACS method according to the manufacturer's protocol (Miltenyi Biotec, the Netherlands). Cell suspensions were labeled with superparamagnetic MicroBeads coupled to antibodies specific for the astrocyte marker GLAST (Anti-GLAST, MicroBead Kit, Miltenyi Biotec). Before antibody labeling, nonspecific binding to the Fc receptor was blocked using the FcR Blocking Reagent (Miltenyi Biotec). Cells were suspended in PBS with 0.5% BSA and the cell suspension was loaded onto an MS Column (Miltenyi Biotec), which was placed in the magnetic field of a MiniMACS Separator (Miltenyi Biotec). The magnetically labeled GLAST-positive cells were retained within the column and eluted as the positively selected cell fraction after removing the column from the magnet. RNA from the sorted cells was extracted and converted to cDNA. qPCR was performed as reported previously [51].

Sequences of primers used for RT-PCR or qPCR (5′ to 3′) were as follows: *Grm3* (PrimerBank ID, 32469489a1): For-CTGGAGGCCATGTTGTTTGC, Rev-CATCCACTTTAGTCAACGATGCT;

*Grm5* (PrimerBank ID, 219801746c1): For-ACCAACCAACTGTGGACAAAG, Rev-CAAGAGTGTGGGATCTGAATTGA; *Ezr* (PrimerBank ID, 83921617c2): For-CACAGGAGGTCCGAAAGGAGA, Rev-CTTGGCCTGAACGGCATAGG; *gapdh* (PrimerBank ID, 126012538c1): For-AGGTCGGTGTGAACGGATTTG, Rev-GGGGTCGTTGATGGCAACA.

### Primary astrocyte culture

Primary astrocyte cultures were prepared from the cerebral cortex of newborn (P0) C57BL/6 mice as described previously [53], with minor modifications. Briefly, the mice were decapitated and the brain structures were removed. Cortexes from three mice were dissected and rinsed in cold Hank’s balanced salt solution (HBSS), and the meninges were carefully stripped off. Tissue was triturated and, after centrifugation, the pellet was resuspended in astrocyte culture medium (DMEM containing 10% fetal bovine serum [Giboco], 0.5% penicillin/streptomycin, and 1% GlutaMAX supplement [Giboco]). Cells were plated in T-75 flasks (Corning) previously coated with poly-L-lysine (Sigma) and incubated at 37°C in a humidified 5% CO_2_, 95% air chamber for 7–8 d until reaching confluence. After growing to confluence, cells were shaken for 7 h at 240 rotations per min (RPM) on an orbital shaker to remove microglia and oligodendrocyte precursor cells. For morphology analysis, purified astrocytes were plated onto poly-D-lysine–coated coverslips at 50,000 cells/well on a 12-well plate before the day of the transfection (at 9–13 in vitro). EGFP plasmids were then transfected into astrocytes using a modified Lipofectamine protocol with Lipofectamine 3000 (Invitrogen) according the manufacturer’s instructions, applied directly to cultured cells and allowing overnight (24-h) expression.

### Immunohistochemical evaluations

Immunohistochemistry (IHC) was performed as previously reported [51]. For immunofluorescence in cultured astrocytes, cells were fixed with 4% paraformaldehyde (15 min, 0°C), rinsed with glycine solution (30 mM in 0.1 M PBS solution), and permeabilized with Triton X-100 (0.2%, 3 min). Preincubation was performed with 10% NGS for 1 h at 37°C. The following primary antibodies were used: mouse anti-NeuN (1:1000; Abcam #ab104224), mouse anti-GFAP (1:500, Millipore #MAB360), rabbit anti-GFAP (1:500, Proteintech #16825-1-AP), guinea pig anti-S100β (1:500, Synaptic Systems #287 004), rabbit anti-Ezrin (1:100, Cell Signaling #3145), mouse anti-vGluT1 (1:200, Millipore #MAB5502), and rabbit anti-PSD95 (1:100, Invitrogen #51–6900). The following Alexa conjugated secondary antibodies were used: goat anti-mouse 488 (1:1,000, Abcam #ab150113), goat anti-rabbit-488 (1:1,000, Abcam #ab150077), goat anti-rabbit 647 (1:500, Abcam #ab150079), and goat anti-guinea pig 488 (1:500, Abcam #ab150185). Immunofluorescence of Ezrin was performed by using an Alexa Fluor 488 Tyramide SuperBoost Kit (Invitrogen #B40912 and #B40941) according to the manufacturer’s protocols. After nucleus labeling with DAPI, the coverslips were mounted on slides using anti-fade solution. For quantification of fluorescent intensity, sections from the two groups were stained and imaged with exactly the same protocol.

### Synaptic counting

After vGluT1 and PSD95 staining, high-magnification 60× objective lens plus 4× optical zoom Z-stack images were obtained using the Nikon A1R^+^ multiphoton microscope. To obtain a high signal-to-noise ratio, ER function of Nikon A1R^+^ multiphoton microscope was further used. Five-micrometer-thick Z-stacks of 20 optical sections were imaged with all three channels: vGluT1 (green), mCherry (red), and PSD95 (magenta), and four consecutive optical sections (thickness of 1 μm) were then extracted and imported to Imaris for further analysis. The number of colocalized synaptic puncta of excitatory intracortical vGluT1/PSD95 was obtained using the Imaris plugin Surface-surface colocalization after 3D reconstructing of vGluT1/PSD95 with the Surface function. For each image, colocalized synaptic puncta were quantified in mCherry^+^ and mCherry^−^ domains of ROIs of 144 μm^3^ (12 by 12 by 1 μm), which were devoid of regions with neuronal cell bodies (areas lacking synaptic puncta). Synaptic puncta quantification was calculated as a density (per 100 μm^3^).

### TUNEL staining

TUNEL assay was performed by using the FragEL DNA Fragmentation Detection Kit (Millipore #QIA39) following the manufacturer’s instructions. After TUNEL staining, sections were then incubated with DAPI-containing mounting media. The 10× objective lens plus 1.5× optical zoom Z-stack images were obtained using the Nikon A1R^+^ multiphoton microscope. Then, the number of TUNEL^+^ and DAPI^+^ cells within the whole image views was quantified by using the Spots function of Imaris. The density (per 1 mm^2^) and percentage (%) of TUNEL^+^ cells were calculated.

### Morris water maze

The Morris water maze was performed on P42–P47 mice. The system consisted of a swimming pool (120-cm diameter) containing white water and an escape platform (10-cm diameter) submerged 0.5 cm under the water surface. The platform remained in the same location during the acquisition phase. Mice were trained in four trials daily for 6 consecutive days. Mice were put into the water maze in random positions facing the wall. They were given 60 s to locate the hidden platform. If they failed to locate within 60 s, they were guided to the platform and allowed to stay there for 15 s. The latency to reach the platform was measured by a video tracking system (Smart, Panlab, Spain). If they failed to locate the hidden platform, the latency was regarded as 65 s. The four daily trials were averaged for each animal. Twenty-four hours after the last training session, the escape platform was removed and the time spent in the target quadrant during a 60-s trial was recorded.

### Fear conditioning test

The P52 mice were subjected to a fear conditioning test. The conditioning trial consisted of a 5-min habituation period followed by six conditioned stimulus (CS)–unconditioned stimulus (US) pairings separated by 1 min each (CS, 80 db white noise, 20-s duration; US, 0.8 mA foot-shock intensity, 1-s duration; US was delivered during the last second of the CS presentation). Twenty-four hours after conditioning, a contextual test was performed in the same chamber for 5 min in the absence of white noise. A cued test was performed 2 h after the contextual test by presenting the cue (80 db white noise) in another chamber with different visual and tactile cues for 3 min. The freezing time (absence of movement in any part of the body) was recorded with ANY-maze video tracking system (Stoelting, Wood Dale, IL).

### Reciprocal social interactions

Reciprocal social interactions were conducted on P27–P30 mice. A pair of mice with same sex and group but from different litters was placed in the testing arena, which was covered with a 0.5-cm layer of clean bedding. Their interactions were video recorded for 30 min. Behavioral events, including following, nose-to-nose sniffing, pushing, or crawling, were scored.

### Three-chamber test

Three-chamber test was conducted on P30–P48 mice. The test was composed of three sessions: habituation, sociability, and social recognition. The mouse was first put into the center chamber and allowed to explore only the center chamber for 5 min during the habituation phase. In the sociability phase, a social stimulus (a stranger mouse with the same strain, gender, and age as the subject mouse) was put into an inverted wire cage and placed in one of the two side chambers, while another same empty cage (nonsocial stimulus) was placed on the other side. The subject mouse was allowed to freely explore the three chambers for 10 min. In the social recognition phase, another novel mouse (novel stimulus) was introduced into the previously empty cage. The subject mouse was also allowed to explore for 10 min in the third phase. Physical contacts with the cages of the subject mice by the nose, head, and forelimbs were defined as sniffing, which was cumulatively scored as total duration in seconds over each 10-min test session.

### Spontaneous activity

At P28–P31, spontaneous activity of Control and Sevo group mice was tested using a spontaneous activity assessment device (TAIMENG, Chengdu, China) equipped with infrared detectors to detect the horizontal and vertical activities. Each subject mouse was placed in one testing chamber, allowing for free activity, and the time of movements (horizontally) and standings (vertically) during the 1-h observation was recorded automatically.

### Rotarod test

The mouse was initially placed on the stationary rod. After habituation, rotation was started at 4 RPM, accelerating over a 5-min period to 40 RPM. The RPM taken for the mouse to fall from the rod was recorded.

### Open field test

Open field test was performed on P35 mice. Mice were placed in the center of a novel open field arena (50 by 38 by 20 cm, white floor) and allowed to freely explore the arena for 10 min. Exploratory behaviors were recorded by a video tracking system (Smart, Panlab, Spain). The total distance traveled, number of entrances to the central area, and the time spent in the central area were recorded.

### Statistics

All statistical analyses were performed using Origin 9 software (OriginLab) or Graphpad prism 7. Data fitting a parametric distribution were tested for significance using analysis of paired and unpaired Student two-tailed *t* tests; data fitting a nonparametric distribution were tested for significance using two-tailed Mann–Whitney. Data with more than two groups were tested for significance using one-way ANOVA test followed by post hoc Tukey test (parametric data) or Kruskal-Wallis test followed by Benjamini, Krieger, and Yekutieli multiple comparison test (nonparametric data). Significance was defined as *P <* 0.05.

## Supporting information

S1 Table Artery blood gas analysis of P7 mice. Data are shown as mean ± SEM, *n* = 7 and *n* = 5 mice in Control and Sevo groups, respectively.
(XLSX)

S1 Fig Early Sevo exposure neither affected astrocyte soma and primary branch morphology nor induced apparent astrogliosis in the somatosensory cortex. (**A**) Representative confocal image and 3D reconstructed soma and primary branches of a cortical astrocyte. Scale bar, 10 μm. (**B**) **Left**, quantification of astrocytic soma volume at P8 (*P* = 0.216, unpaired *t* test), P14 (*P* = 0.652, unpaired *t* test), and P21 (*P* = 0.449, unpaired *t* test); **middle**, quantification of primary branches volume of astrocytes in the somatosensory cortex at P8 (*P* = 0.098, unpaired *t* test), P14 (*P* = 0.826, unpaired *t* test), and P21 (*P* = 0.798, unpaired *t* test); **right**, average astrocytes number of primary branches in the somatosensory cortex at P8 (*P* = 0.592, Mann-Whitney test), P14 (*P* = 0.219, Mann-Whitney test), and P21 (*P* = 0.065, Mann-Whitney test). (**C**) Representative confocal images (left), zoom in (right top), and 3D reconstruction (right bottom) of GFAP in the somatosensory cortex at P14. Scale bars, 100 μm (left), 10 μm (right). (**D**) Quantification of GFAP volume (*P* = 0.263, unpaired *t* test) (**left**) and GFAP^+^ cells density (*P* = 0.731, unpaired *t* test) (**right**). Data are shown as mean ± SEM. Underlying data are available in S1 Data. GFAP, glial fibrillary acidic protein; n.s., not significant; Sevo, sevoflurane.
(TIF)

S2 Fig Early lengthy Sevo exposure did not lead to astrocyte morphological deficits in the hippocampal CA1sr and DG-mo at P14. (**A)** Diagram of hippocampal CA1sr and DG-mo. (**B**) Representative fluorescent images and distal fine processes reconstructions of astrocytes in the hippocampal CA1sr and DG-mo of P14 mice. Scale bars, 10 μm. (**C, D**) Average distal and proximal fine processes volume of astrocytes in the hippocampal CA1sr and DG-mo of P14 mice (CA1sr distal fine processes: *P* = 0.888; DG-mo distal fine processes: *P* = 0.278; CA1sr proximal fine processes: *P* = 0.009; DG-mo proximal fine processes: *P* = 0.046, unpaired *t* test). (**E, F, G, H**) Quantification of astrocytic territory (**E**) and soma (**F**) volume, primary branches volume (**G**), and number of primary branches (**H**), respectively (CA1sr territory volume: *P* = 0.737; DG-mo territory volume: *P* = 0.20; CA1sr soma volume: *P* = 0.895; DG-mo soma volume: *P* = 0.347; CA1sr primary branches volume: *P* = 0.553; DG-mo number of primary branches: *P* = 0.797, unpaired *t* test; DG-mo primary branches volume: *P* = 0.037; CA1sr number of primary branches: *P* = 0.905, Mann-Whitney test). *P < 0.05; **P < 0.01; n.s., not significant. Data are shown as mean ± SEM. Underlying data are available in S1 Data. CA1sr, CA1 stratum radiatum; DG-mo, molecular layer of dentate gyrus; n.s., not significant; Sevo, sevoflurane.
(TIF)

S3 Fig One-hour Sevo exposure to P7 mice or 4-h exposure to P42–P50 mice did not impair astrocyte morphogenesis. (**A**) Experiment protocol for Sevo exposure and morphological assessment. (**B)** Fluorescent images and reconstructed distal fine processes of astrocytes. Scale bars, 10 μm. (**C, D**) Quantification of astrocytic distal and proximal fine processes volume (P42–P50 distal fine processes: *P* = 0.30, unpaired *t* test; P14 distal fine processes: *P* = 0.486, Mann-Whitney test; P42–P50 proximal fine processes: *P* = 0.915; P14 proximal fine processes: *P* = 0.552, unpaired *t* test). (**E, F, G, H)** Quantification of astrocytic territory **(E)** and soma **(F)** volume, volume **(G)** and number **(H)** of primary branches, respectively (P42–P50 soma volume: *P* = 0.173; P14 soma volume: *P* = 0.391; P42–P50 territory volume: *P* = 0.268; P14 territory volume: *P* = 0.439, unpaired *t* test; P42–P50 primary branches volume: *P* = 0.675; P14 primary branches volume: *P* = 0.187; P42–P50 number of primary branches: *P* = 0.978; P14 number of primary branches: *P* = 0.181, Mann-Whitney test). Data are shown as mean ± SEM. Underlying data are available in S1 Data. n.s., not significant; Sevo, sevoflurane.
(TIF)

S4 Fig Apoptosis assays in Sevo and Control group mice at P8, and unaltered total and mushroom apical dendritic spine density in the hippocampal CA1sr at P21. (**A, B**) Confocal images of TUNEL staining in the cortex of Control and Sevo mice at P8. Scale bar, 1,000 μm in **A**, 100 μm in **B**. (**C, D**) Quantification of the density ([**C**] *P* = 0.966, Mann Whitney test) and percentage ([**D**] *P* = 0.989, Mann Whitney test) of TUNEL-positive (TUNEL^+^) cells. (**E**) Confocal images of apical dendritic spines in the hippocampal CA1sr. Scale bar, 2 μm. (**F**) Quantification of total (*P* = 0.079, unpaired *t* test) and mushroom (*P* = 0.267, unpaired *t* test) apical dendritic spine density. Data are shown as mean ± SEM. Underlying data are available in S1 Data. CA1sr, CA1 stratum radiatum; n.s., not significant; TUNEL, terminal deoxynucleotidyl transferase deoxyuridine triphosphate nick-end labeling.
(TIF)

S5 Fig eEPSCs pharmacological blockade, input resistance, and decay kinetics in cortical pyramidal neurons from Control and Sevo group mice. (**A**) Traces depicting pharmacological eEPSCs at −60 mV and +40 mV. (**B**) Input resistance of pyramidal neurons in Control and Sevo groups (*P* = 0.154, unpaired *t* test) (**C, D**) Quantification of the decay kinetics (weighted time constants) of AMPAR-mediated eEPSCs and NMDAR-mediated eEPSCs in Control and Sevo group mice ([**C**] *P* = 0.167, Mann-Whitney test; [**D**] *P* = 0.119, Mann-Whitney test). **P < 0.01; n.s., not significant. Data are shown as mean ± SEM. Underlying data are available in S1 Data. AMPAR, α-amino-3-hydroxy-5-methyl-4-isoxazole propionate receptor; eEPSC, evoked excitatory postsynaptic current; NMDAR, N-methyl-D-aspartic acid receptor; n.s., not significant.
(TIF)

S6 Fig Lengthy Sevo exposure did not affect the cognitive functions, locomotive ability, or sociability of mice. **(A) Left**, latencies to locate the escape platform during the acquisition phase in Morris water maze on P42–P47 mice (day 1: *P* = 0.124, Mann-Whitney test; day 2: *P* = 0.090, unpaired *t* test; day 3: *P* = 0.623, unpaired *t* test; day 4: *P* = 0.031, Mann-Whitney test; day 5: *P* = 0.614, unpaired *t* test; day 6: *P* = 0.246, unpaired *t* test). **Right**, time in the target quandrant during the 60-s probe test (*P* = 0.887, unpaired *t* test). (**B**) Freezing time in the preconditioning phase, contextual and cue test from Control and Sevo groups at P52–P57 (preconditioning: *P* = 0.593, unpaired *t* test; contextual: *P* = 0.903, Mann-Whitney test; cue: *P* = 0.714, Mann-Whitney test). (**C**) **Left**, cartoon illustrating the three-chamber test (sociability) at P32–P48. **Right**, quantification of time spent sniffing the mouse (social) and empty (nonsocial) in Control and Sevo groups (Control: *P* < 0.001; Sevo: *P* < 0.001; Mann-Whitney test). (**D**) Quantification of spontaneous activity in Control and Sevo groups at P28–P31 (movements: *P* = 0.150; standings: *P* = 0.693; Mann-Whitney test). (**E**) The RPM taken for the mice to fall from the rod from Control and Sevo groups at P56 in Rotarod test (*P* = 0.632; Mann-Whitney test). (**F**) Number of entrances to the central area (**left**, *P* = 0.565; Mann-Whitney test), time spent in the central area (**middle**, *P* = 0.346; Mann-Whitney test), and total distance traveled (**right**, *P* = 0.893, unpaired *t* test) in the open field test in Control and Sevo groups at P35. *P < 0.05; ***P < 0.001; n.s., not significant. Data are shown as mean ± SEM. Underlying data are available in S1 Data. n.s., not significant; RPM, rotations per min; Sevo, sevoflurane.
(TIF)

S7 Fig Stereotactic injection of AAV5·gfaABC_1_D·GCaMP6f into the cortex of P0 mice did not induce apparent astrogliosis at P14. Fluorescent images of auto-EGFP (green) and GFAP (red) in the cortex of WT and GCaMP6f-injected mice at P14. Scale bars, 200 μm. EGFP, enhanced green fluorescent protein; GCaMP6f, AAV5•gfaABC_1_D•GCaMP6f; GFAP, glial fibrillary acidic protein; WT, wild-type.
(TIF)

S8 Fig Inhibition of basal Ca^2+^ level and spontaneous Ca^2+^ signals following acute Sevo exposure. (**A**) Quantification of acute Sevo application on cortical astrocyte basal Ca^2+^ from adult mice at P56 (*P* = 0.004, unpaired *t* test). (**B**) Quantification of the two types of spontaneous Ca^2+^ signals properties, including frequency, peak amplitude, and half-width, before and after acute Sevo exposure (*P* = 0.009, paired *t* test, for waves frequency; *P* = 0.997, paired *t* test for microdomains frequency; *P* = 0.009, Mann-Whitney test for waves peak amplitude; *P* = 0.188, Mann-Whitney test for microdomains peak amplitude; *P* = 0.717, Mann-Whitney test for waves half-width; *P* = 0.742, Mann-Whitney test for microdomains half-width). Data are shown as mean ± SEM. Underlying data are available in S1 Data. n.s., not significant; Sevo, sevoflurane.
(TIF)

S9 Fig Unchanged Ezrin expression levels in the hippocampal CA1sr and DG-mo in Sevo group mice at P14. (**A**) Representative fluorescent images in the hippocampus of Control and Sevo group mice at P14. Scale bars, 200 μm. (**B**) Quantification of Ezrin fluorescent intensity in the hippocampal CA1sr (*n* = 96 ROIs from 6 mice in the Control group, *n* = 86 ROIs from 6 mice in the Sevo group; *P* = 0.295, Mann-Whitney test) and DG-mo (*n* = 84 ROIs from 6 mice in the Control group, *n* = 76 ROIs from 6 mice in the Sevo group; *P* = 0.164, Mann-Whitney test). Data are shown as mean ± SEM. Underlying data are available in S1 Data. CA1sr, CA1 stratum radiatum; DG-mo, molecular layer of dentate gyrus; n.s., not significant; ROI, region of interest.
(TIF)

S10 Fig Ezrin-miRNAi, with high KD efficiency and cell-type specificity, led to increased astrocyte soma volume but unchanged primary branches morphology in the cortex at P21. (**A**) Fluorescent images of Ezrin and mCherry in the cortex of NC-miRNAi and Ezrin-miRNAi mice at P21. Scale bars, 100 μm. (**B**) Quantification of fold change in Ezrin fluorescent intensity, measured as (F_mCherry+_ − F_mCherry−_)/ F_mCherry−_ (*P* = 0, Mann-Whitney test). (**C**) Schematic fluorescent images of S100β, mCherry, and NeuN in the cortex of Ezrin-miRNAi–injected mice at P21. Scale bars, 50 μm. (**D**) Quantification of mCherry^+^ cells colocalized with S100β and NeuN. (**E**) Images of GFAP and mCherry in the cortex of Ezrin-miRNAi and NC-miRNAi–injected mice at P21. Scale bars, 20 μm. (**F**) Confocal image with 3D reconstructed soma and primary branches of mCherry-labeled astrocyte. Scale bar, 10 μm. (**G**) Quantification of astrocyte soma volume (**left**), primary branches volume (**middle**), and number of primary branches (**right**), respectively, in NC-miRNAi and Ezrin-miRNAi–injected mice (soma volume: *P* < 0.001, number of primary branches: *P* = 0.095, Mann-Whitney test; primary branches volume: *P* = 0.072, unpaired *t* test). ***P < 0.001; n.s., not significant. Data are shown as mean ± SEM. Underlying data are available in S1 Data. GFAP, glial fibrillary acidic protein; KD, knock-down; miRNAi, microRNA-based RNA interference; NC, negative control; NeuN, neuronal nuclei; n.s., not significant.
(TIF)

S11 Fig Ezr was widely expressed in the cortex of mice with high cell-type specificity. (**A**) The autofluorescence of mCherry showed that Ezr (AAV8·gfaABC_1_D·Ezrin·P2A·mCherry) was widely expressed in the brain, especially the cortex, of mice at P9, P25, and P40. Scale bars, 1,000 μm. (**B**) Fluorescent images of Ezrin staining (green) and mCherry (red) in the cortex of mCh (AAV8-gfaABC_1_D- mCherry) and Ezr-injected mice at P14. Scale bars, 50 μm. (**C**) Quantification of fold change in Ezrin fluorescent intensity, measured as (F_mCherry+_ − F_mCherry−_)/ F_mCherry−_ (*P* < 0.001, Mann-Whitney test). (**D**) Fluorescent images of NeuN (magenta), mCherry (red), and S100β (green) in the cortex of Ezr-injected mice. Scale bars, 50 μm. (**E**) Quantification of mCherry^+^ cells colocalized with S100β and NeuN. ***P < 0.001. Data are shown as mean ± SEM. Underlying data are available in S1 Data. Ezr, AAV8•gfaABC_1_D•Ezrin•P2A•mCherry; mCh, AAV8•gfaABC_1_D•mCherry; NeuN, neuronal nuclei.
(TIF)

S12 Fig Autofluorescence of mCherry was insufficient to map astrocytic fine processes at P14, and lengthy Sevo exposure did not affect astrocytic soma and primary branches morphology. (**A**) Confocal images of astrocytes labeled with Lucifer yellow (green) and mCherry (red) in the cortex of mCh- and Ezr-injected mice at P14, in which mCherry was insufficient to mark astrocytic fine processes. Scale bars, 10 μm. (**B**) Representative confocal image, 3D reconstructed soma and primary branches of a cortical astrocyte. Scale bar, 10 μm. (**C**) Quantification of the soma volume of cortical astrocytes in mice from Control-mCh, Sevo-mCh, and Sevo-Ezr groups (*P* = 0.894, Kruskal-Wallis test). (**D**, **E**) Quantification of astrocytic primary branches volume (**D**) and number (**E**) in the cortex of mice from Control-mCh, Sevo-mCh, and Sevo-Ezr groups (volume: *P* = 0.385; number: *P* = 0.599; Kruskal-Wallis test). Data are shown as mean ± SEM. Underlying data are available in S1 Data. Ezr, AAV8•gfaABC_1_D•Ezrin•P2A•mCherry; mCh, AAV8•gfaABC_1_D•mCherry; n.s., not significant; Sevo, sevoflurane.
(TIF)

S13 Fig Lengthy Sevo exposure led to synaptic overgrowth, which was not restored in Ezr-negative area. **Characterization of the input resistance, decay kinetics of AMPAR/NMDAR-mediated eEPSCs, and sociability in the three groups of mice**. (**A**) Fluorescent image of vGluT1 (green) and PSD95 (magenta) in mCherry-positive (mCherry^+^) and mCherry-negative (mCherry^−^) area. Scale bar, 5 μm. (**B**) Zoomed-in images of vGluT1 and PSD95 in mCherry^−^ area from the cortex of Control-mCh, Sevo-mCh, and Sevo-Ezr groups. Scale bar, 2 μm. (**C**) Colocalization of vGluT1 and PSD95; the white arrows showed vGluT1/PSD95 colocalized puncta. Scale bar, 2 μm. (**D**) Quantification of vGluT1/PSD95 colocalized puncta in mCherry^−^ area (Control-mCh versus Sevo-mCh: *P* < 0.001; Control-mCh versus Sevo-Ezr: *P* < 0.001; Sevo-mCh versus Sevo-Ezr: *P* = 0.511; Kruskal-Wallis test followed by post hoc multiple comparison test). (**E**) Input resistance of pyramidal neurons in Control-mCh, Sevo-mCh, and Sevo-Ezr groups (*P* = 0.379, one-way ANOVA). (**F**) Quantification of the decay kinetics (weighted time constants) of AMPAR-mediated eEPSCs in Control-mCh, Sevo-mCh, and Sevo-Ezr groups (Control-mCh versus Sevo-mCh: *P* = 0.099; Sevo-mCh versus Sevo-Ezr: *P* = 0.195; Control-mCh versus Sevo-Ezr: *P* = 0.004; Kruskal-Wallis test followed by post hoc multiple comparison test). (**G**) Quantification of the decay kinetics (weighted time constants) of NMDAR-mediated eEPSCs in Control-mCh, Sevo-mCh, and Sevo-Ezr groups (*P* = 0.124, Kruskal-Wallis test). (**H**) Quantification of time spent sniffing the mouse (social) and empty (nonsocial) in three-chamber sociability test at P30–P35 (Control-mCh: *P* < 0.001, paired *t* test; Sevo-mCh: *P* = 0.047, paired *t* test; Sevo-Ezr: *P* < 0.001, Mann-Whitney test). *P < 0.05; *** *P* < 0.001; n.s., not significant. Data are shown as mean ± SEM. Underlying data are available in S1 Data. AMPAR, α-amino-3-hydroxy-5-methyl-4-isoxazole propionate receptor; eEPSC, evoked excitatory postsynaptic current; Ezr, AAV8•gfaABC1D•Ezrin•P2A•mCherry; mCh, AAV8•gfaABC_1_D•mCherry; NMDAR, N-methyl-D-aspartic acid receptor; n.s., not significant; PSD95, postsynaptic density protein 95; Sevo, sevoflurane; vGluT1, vesicular glutamate transporter 1.
(TIF)

S1 Data Data underlying Figs 1–6, S1–S6 Figs and S8–S13 Figs. (XLSX)

## Abbreviations

AAV: adeno-associated virus
AIDN: anesthetic-induced developmental neurotoxicity
AMPAR: α-amino-3-hydroxy-5-methyl-4-isoxazole propionate receptor
BAPTA-AM: 1,2-bis-(o-aminophenoxy)-ethane-N,N,N0 N0-tetraacetic acid, tetra-acetoxymethyl ester
BDNF: brain-derived neurotrophic factor
CA1sr: CA1 stratum radiatum
CNQX: 6-cyano-7-nitroquinoxaline-2,3-dione
CS: conditioned stimulus
DG-mo: molecular layer of dentate gyrus
DHPG: (RS)-3,5-Dihydroxyphenylglycine
DL-AP5: DL-2-Amino-5-phosphonopentanoic acid
DMEM: Dulbecco’s minimum essential medium
eEPSC: evoked excitatory postsynaptic current
EGFP: enhanced green fluorescent protein
ERM: Ezrin, Radixin, and Moesin
Ezr: AAV8•gfaABC_1_D•Ezrin•P2A•mCherry
Ezrin-miRNAi: AAV8•gfaABC_1_D•mCherry•Ezrin-miRNAi
FDA: United States Food and Drug Administration
GA: general anesthetic
GAS: General Anaesthesia compared to Spinal anaesthesia
GCaMP6f: AAV5•gfaABC_1_D•GCaMP6f
GFAP: glial fibrillary acidic protein
GLAST: glutamate-aspartate transporter
*Grm3* or mGluR3: metabotropic glutamate receptor 3
*Grm5* or mGluR5: metabotropic glutamate receptor 5
HBSS: Hank’s balanced salt solution
HD: Huntington disease
IHC: immunohistochemistry
ITR: inverted terminal repeat
KD: knock-down
L3-5: layer 3–5
LY354740: (1S,2S,5R,6S)-2-Aminobicyclo[3.1.0]hexane-2,6-dicarboxylic acid
MACS: magnetic cell sorting
MASK: Mayo Anesthesia Safety in Kids
mCh: AAV8•gfaABC_1_D•mCherry
MEGF10: multiple epidermal growth factor-like domains 10
mEPSCs: miniature EPSCs
MERTK: c-Mer tyrosine kinase receptor
miRNAi: microRNA-based RNA interference
NC-miRNAi: AAV8•gfaABC_1_D•mCherry•NC-miRNAi
NCX: sodium–calcium exchanger
NE: noradrenaline
NeuN: neuronal nuclei
NMDAR: N-methyl-D-aspartic acid receptor
P7: postnatal day 7
PANDA: Pediatric Anesthesia Neurodevelopment Assessment
PB: phosphate buffer
POCD: postoperative cognitive dysfunction
PSD: postsynaptic density
PSD95: postsynaptic density protein 95
ROI: region of interest
RPM: rotations per min
RT-qPCR: quantitative reverse transcription PCR
Sevo: sevoflurane
SBF-SEM: serial block face scanning electron microscopy
TTX: tetrodotoxin
US: unconditioned stimulus
vGluT1: vesicular glutamate transporter 1
WT: wild-type
