# Supplementary figures and images for: Astroglial dysfunctions drive aberrant synaptogenesis and social behavioral deficits in mice with neonatal exposure to lengthy general anesthesia
Source: PLoS Biol. 2019 Aug 21;17(8):e3000086. doi: 10.1371/journal.pbio.3000086 (PMC6719896; doi:10.1371/journal.pbio.3000086)

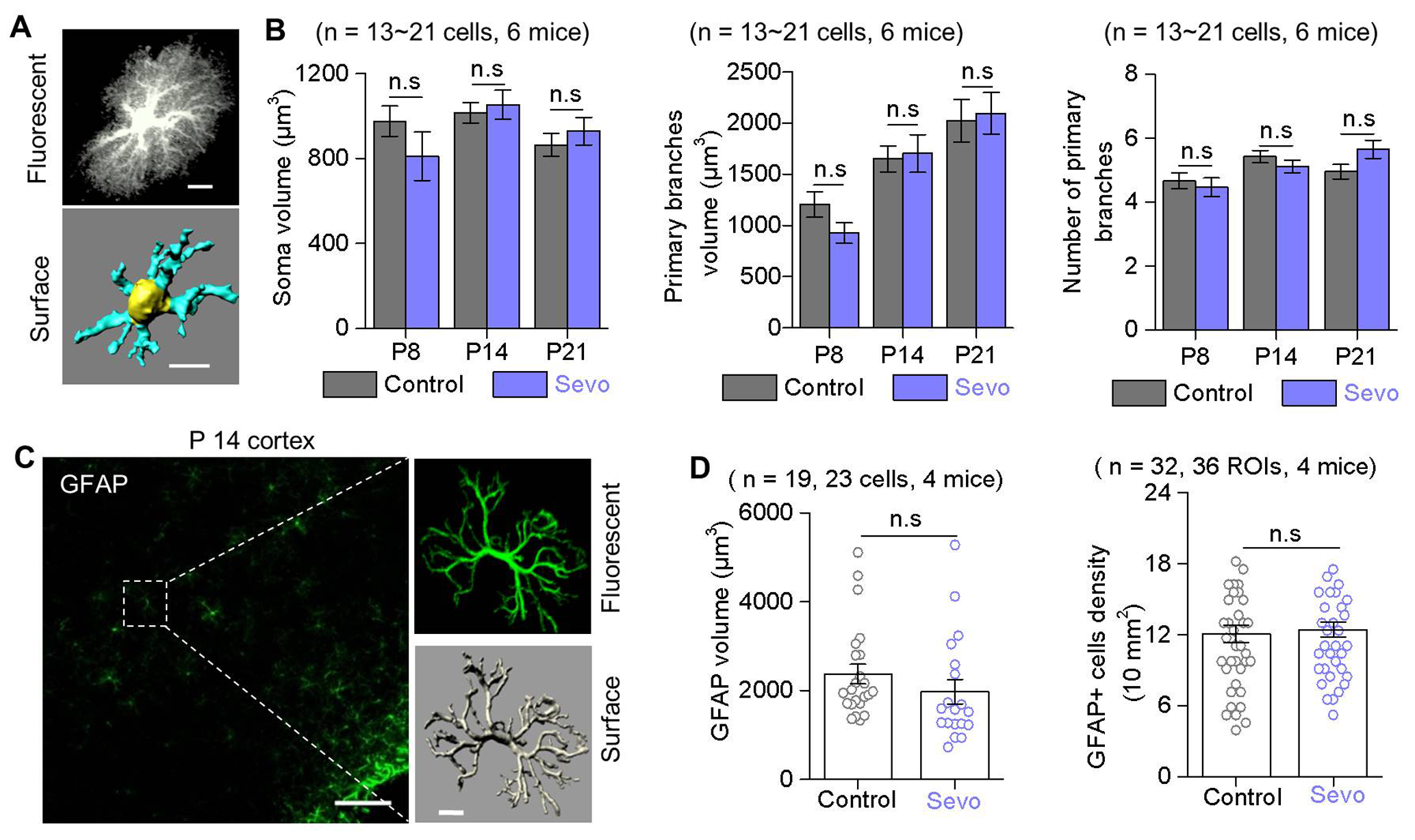

Supplement: S1 Fig — (A) Representative confocal image and 3D reconstructed soma and primary branches of a cortical astrocyte. Scale bar, 10 μm. (B) Left, quantification of astrocytic soma volume at P8 (P = 0.216, unpaired t test), P14 (P = 0.652, unpaired t test), and P21 (P = 0.449, unpaired t test); middle, quantification of primary branches volume of astrocytes in the somatosensory cortex at P8 (P = 0.098, unpaired t test), P14 (P = 0.826, unpaired t test), and P21 (P = 0.798, unpaired t test); right, average astrocytes number of primary branches in the somatosensory cortex at P8 (P = 0.592, Mann-Whitney test), P14 (P = 0.219, Mann-Whitney test), and P21 (P = 0.065, Mann-Whitney test). (C) Representative confocal images (left), zoom in (right top), and 3D reconstruction (right bottom) of GFAP in the somatosensory cortex at P14. Scale bars, 100 μm (left), 10 μm (right). (D) Quantification of GFAP volume (P = 0.263, unpaired t test) (left) and GFAP+ cells density (P = 0.731, unpaired t test) (right). Data are shown as mean ± SEM. Underlying data are available in S1 Data. GFAP, glial fibrillary acidic protein; n.s., not significant; Sevo, sevoflurane. (TIF) [file pbio.3000086.s002.tif]

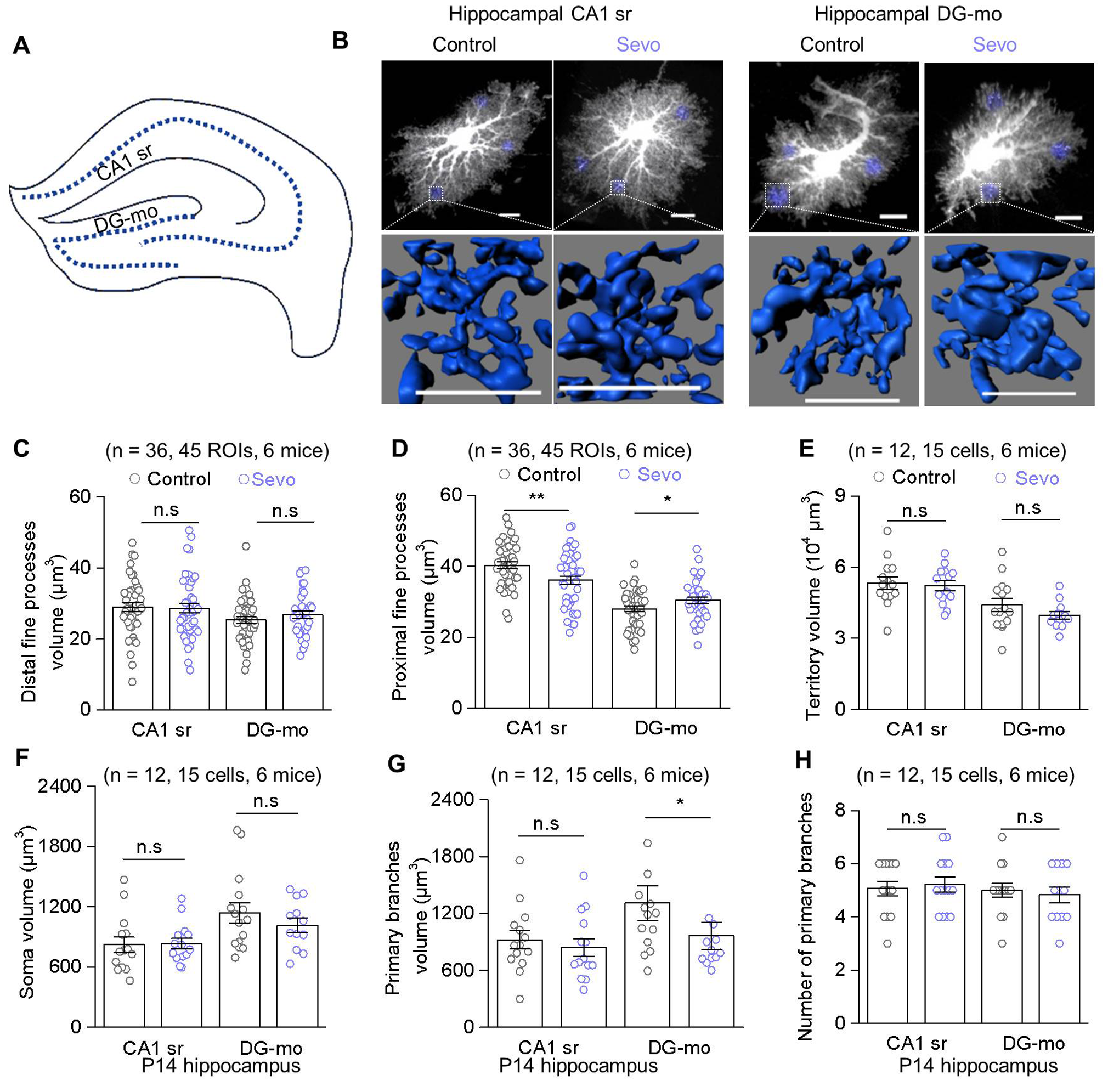

Supplement: S2 Fig — (A) Diagram of hippocampal CA1sr and DG-mo. (B) Representative fluorescent images and distal fine processes reconstructions of astrocytes in the hippocampal CA1sr and DG-mo of P14 mice. Scale bars, 10 μm. (C, D) Average distal and proximal fine processes volume of astrocytes in the hippocampal CA1sr and DG-mo of P14 mice (CA1sr distal fine processes: P = 0.888; DG-mo distal fine processes: P = 0.278; CA1sr proximal fine processes: P = 0.009; DG-mo proximal fine processes: P = 0.046, unpaired t test). (E, F, G, H) Quantification of astrocytic territory (E) and soma (F) volume, primary branches volume (G), and number of primary branches (H), respectively (CA1sr territory volume: P = 0.737; DG-mo territory volume: P = 0.20; CA1sr soma volume: P = 0.895; DG-mo soma volume: P = 0.347; CA1sr primary branches volume: P = 0.553; DG-mo number of primary branches: P = 0.797, unpaired t test; DG-mo primary branches volume: P = 0.037; CA1sr number of primary branches: P = 0.905, Mann-Whitney test). *P < 0.05; **P < 0.01; n.s., not significant. Data are shown as mean ± SEM. Underlying data are available in S1 Data. CA1sr, CA1 stratum radiatum; DG-mo, molecular layer of dentate gyrus; n.s., not significant; Sevo, sevoflurane. (TIF) [file pbio.3000086.s003.tif]

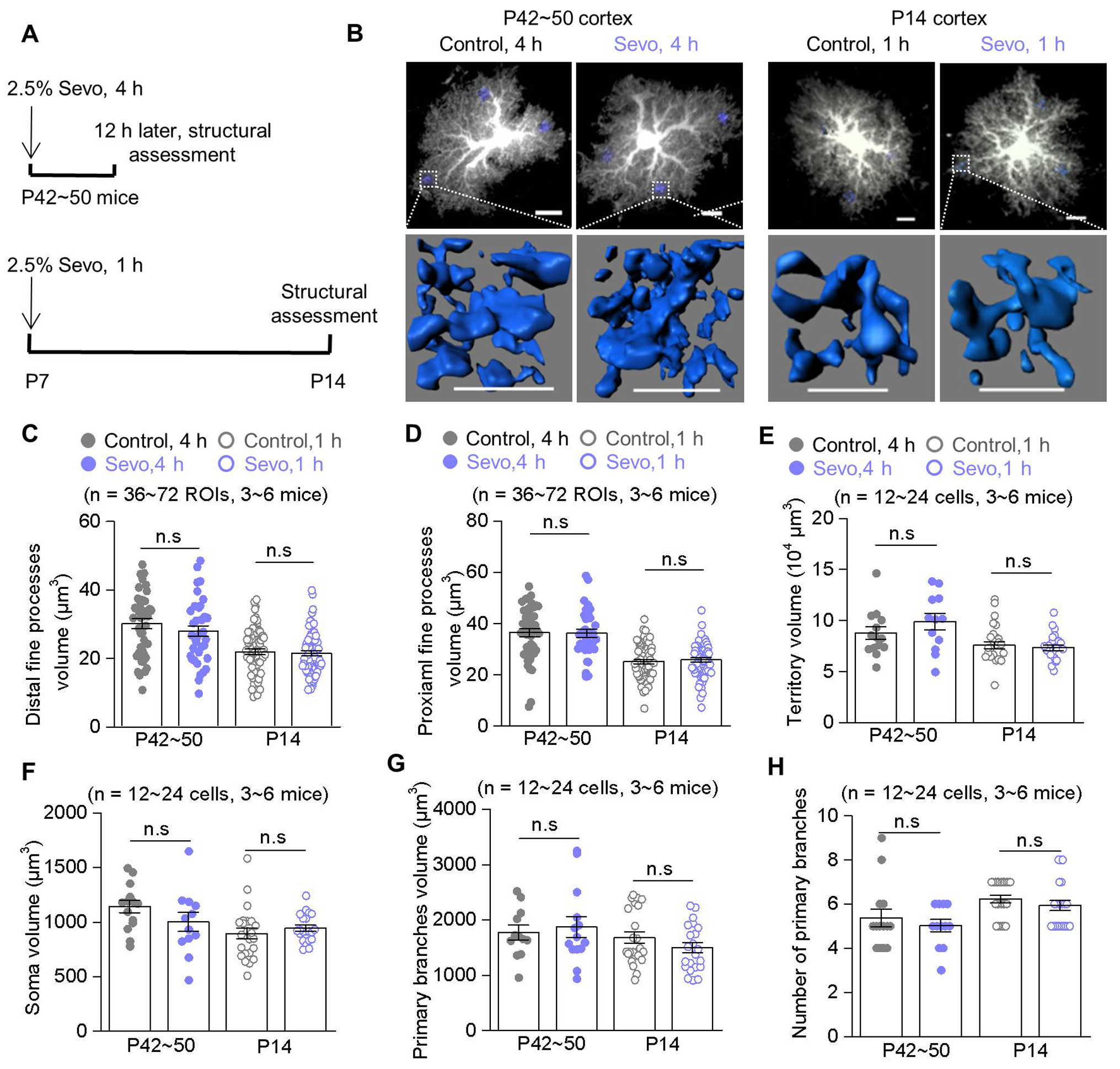

Supplement: S3 Fig — (A) Experiment protocol for Sevo exposure and morphological assessment. (B) Fluorescent images and reconstructed distal fine processes of astrocytes. Scale bars, 10 μm. (C, D) Quantification of astrocytic distal and proximal fine processes volume (P42–P50 distal fine processes: P = 0.30, unpaired t test; P14 distal fine processes: P = 0.486, Mann-Whitney test; P42–P50 proximal fine processes: P = 0.915; P14 proximal fine processes: P = 0.552, unpaired t test). (E, F, G, H) Quantification of astrocytic territory (E) and soma (F) volume, volume (G) and number (H) of primary branches, respectively (P42–P50 soma volume: P = 0.173; P14 soma volume: P = 0.391; P42–P50 territory volume: P = 0.268; P14 territory volume: P = 0.439, unpaired t test; P42–P50 primary branches volume: P = 0.675; P14 primary branches volume: P = 0.187; P42–P50 number of primary branches: P = 0.978; P14 number of primary branches: P = 0.181, Mann-Whitney test). Data are shown as mean ± SEM. Underlying data are available in S1 Data. n.s., not significant; Sevo, sevoflurane. (TIF) [file pbio.3000086.s004.tif]

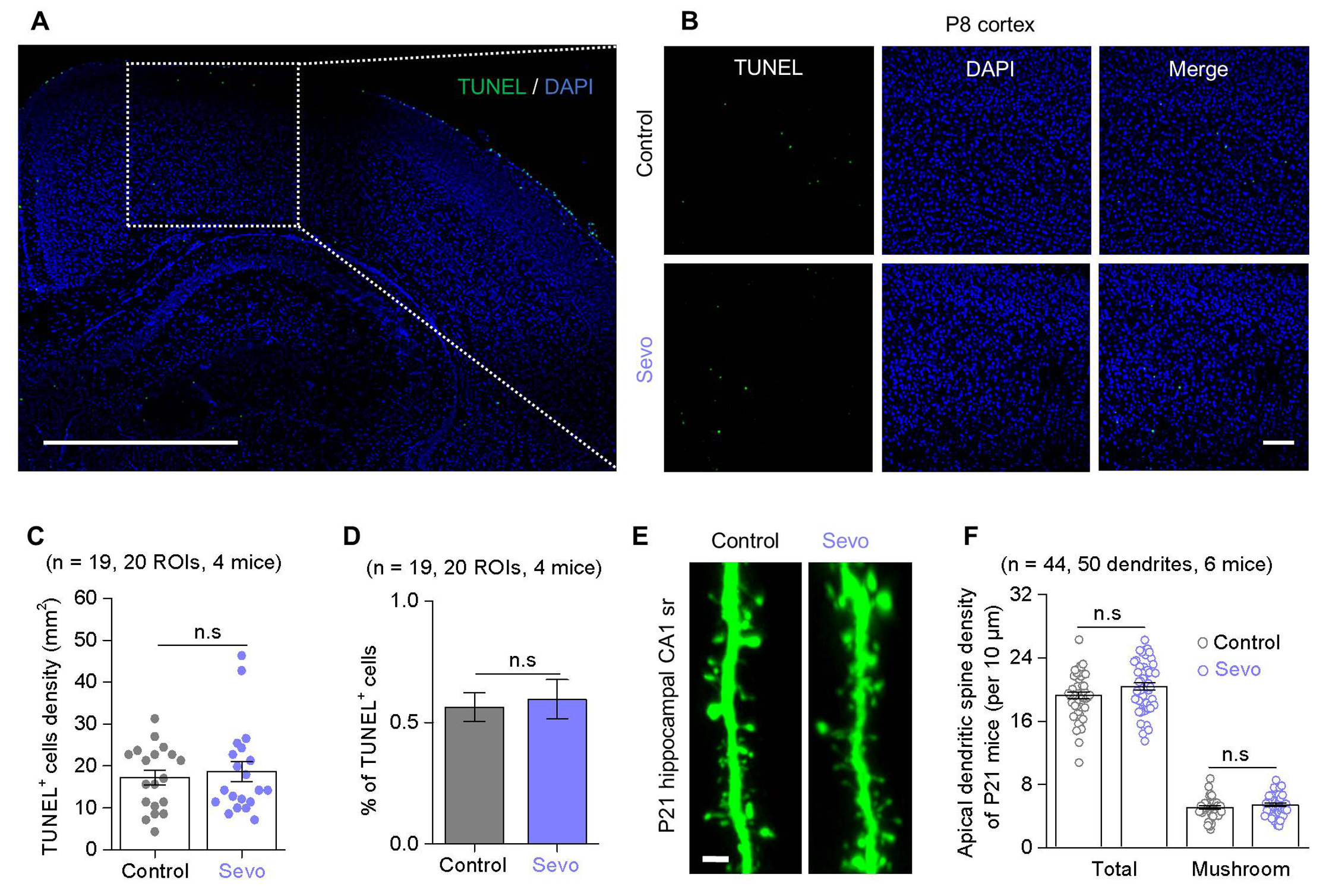

Supplement: S4 Fig — (A, B) Confocal images of TUNEL staining in the cortex of Control and Sevo mice at P8. Scale bar, 1,000 μm in A, 100 μm in B. (C, D) Quantification of the density ([C] P = 0.966, Mann Whitney test) and percentage ([D] P = 0.989, Mann Whitney test) of TUNEL-positive (TUNEL+) cells. (E) Confocal images of apical dendritic spines in the hippocampal CA1sr. Scale bar, 2 μm. (F) Quantification of total (P = 0.079, unpaired t test) and mushroom (P = 0.267, unpaired t test) apical dendritic spine density. Data are shown as mean ± SEM. Underlying data are available in S1 Data. CA1sr, CA1 stratum radiatum; n.s., not significant; TUNEL, terminal deoxynucleotidyl transferase deoxyuridine triphosphate nick-end labeling. (TIF) [file pbio.3000086.s005.tif]

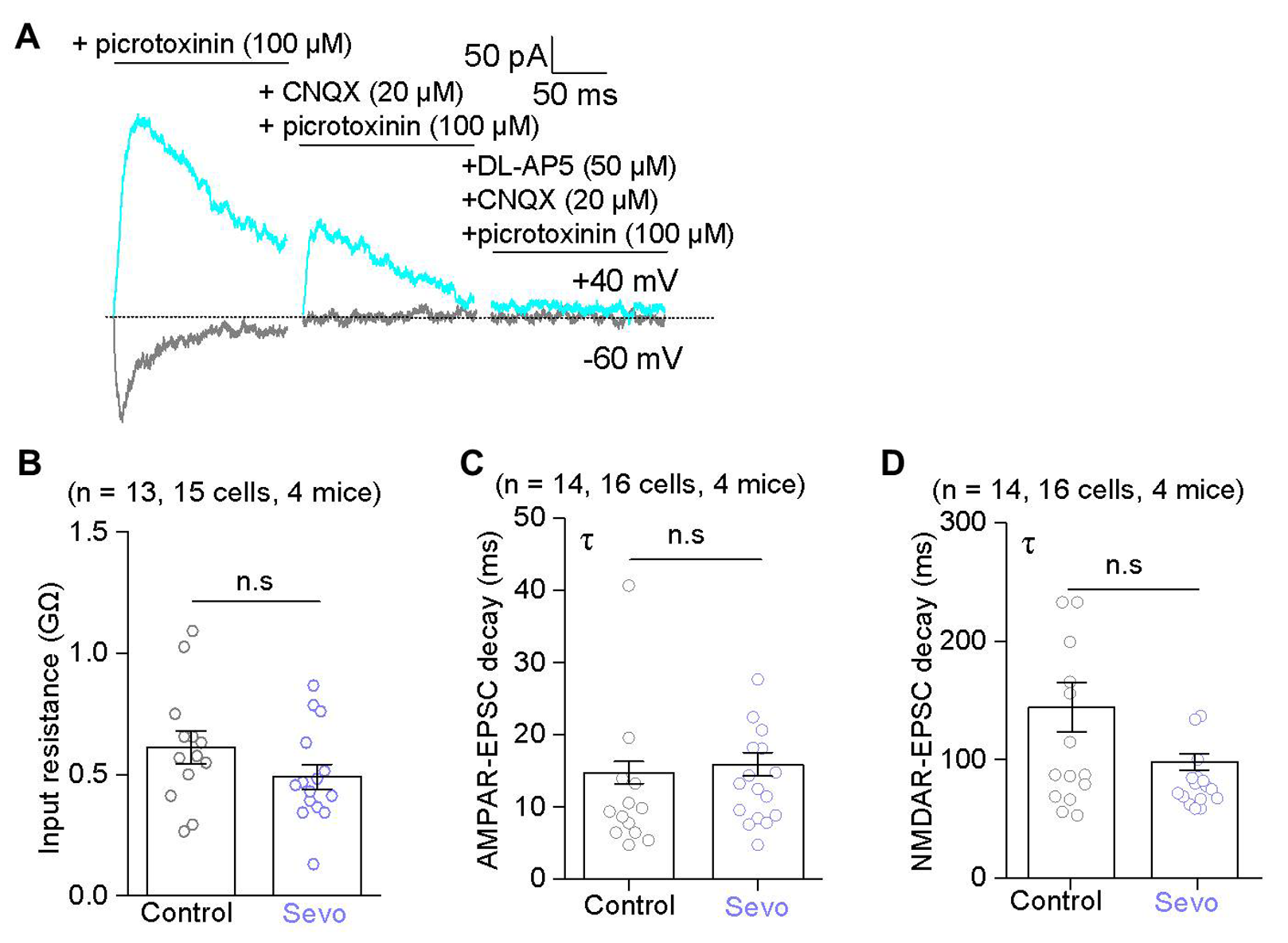

Supplement: S5 Fig — (A) Traces depicting pharmacological eEPSCs at −60 mV and +40 mV. (B) Input resistance of pyramidal neurons in Control and Sevo groups (P = 0.154, unpaired t test) (C, D) Quantification of the decay kinetics (weighted time constants) of AMPAR-mediated eEPSCs and NMDAR-mediated eEPSCs in Control and Sevo group mice ([C] P = 0.167, Mann-Whitney test; [D] P = 0.119, Mann-Whitney test). **P < 0.01; n.s., not significant. Data are shown as mean ± SEM. Underlying data are available in S1 Data. AMPAR, α-amino-3-hydroxy-5-methyl-4-isoxazole propionate receptor; eEPSC, evoked excitatory postsynaptic current; NMDAR, N-methyl-D-aspartic acid receptor; n.s., not significant. (TIF) [file pbio.3000086.s006.tif]

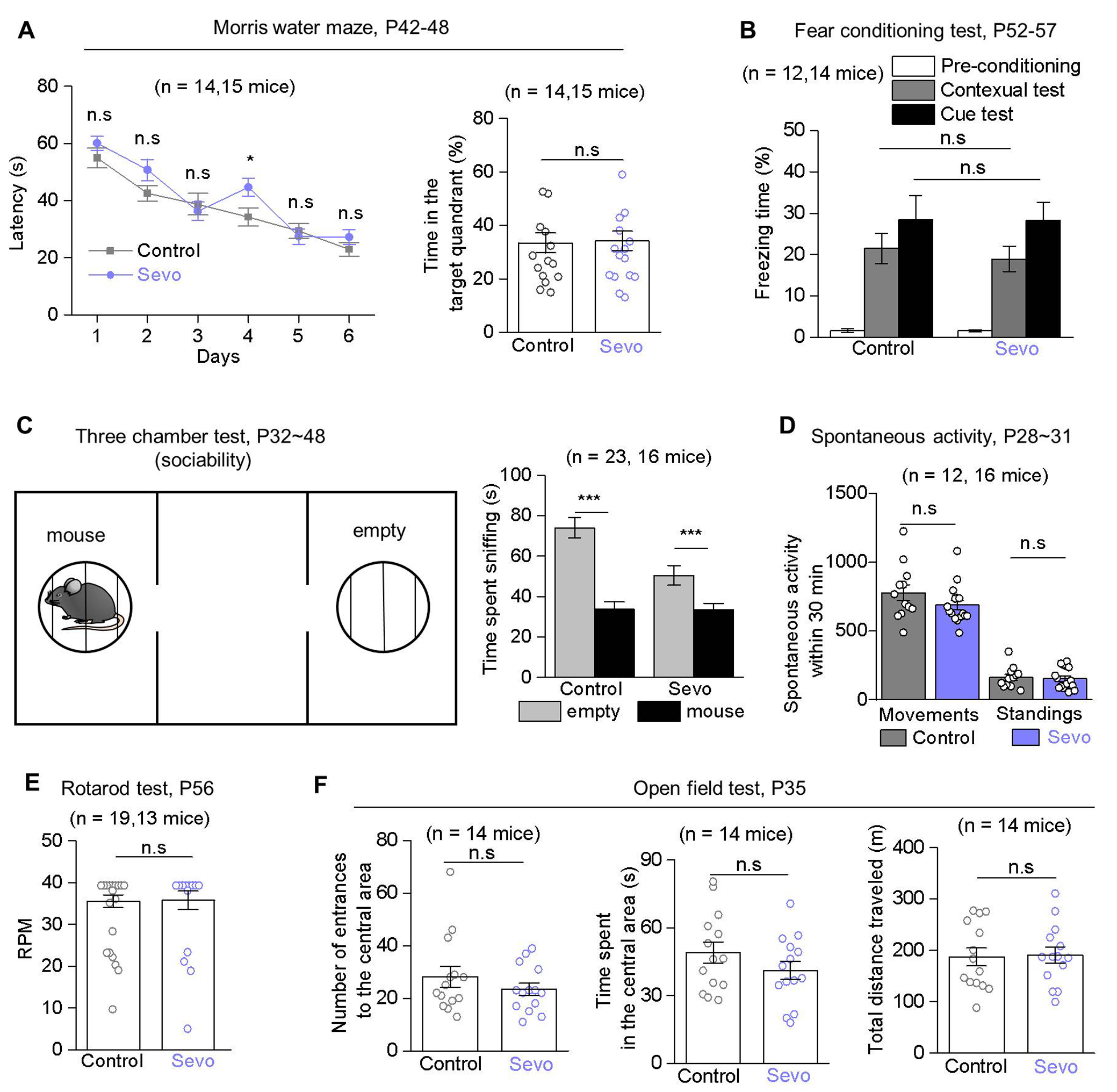

Supplement: S6 Fig — (A) Left, latencies to locate the escape platform during the acquisition phase in Morris water maze on P42–P47 mice (day 1: P = 0.124, Mann-Whitney test; day 2: P = 0.090, unpaired t test; day 3: P = 0.623, unpaired t test; day 4: P = 0.031, Mann-Whitney test; day 5: P = 0.614, unpaired t test; day 6: P = 0.246, unpaired t test). Right, time in the target quandrant during the 60-s probe test (P = 0.887, unpaired t test). (B) Freezing time in the preconditioning phase, contextual and cue test from Control and Sevo groups at P52–P57 (preconditioning: P = 0.593, unpaired t test; contextual: P = 0.903, Mann-Whitney test; cue: P = 0.714, Mann-Whitney test). (C) Left, cartoon illustrating the three-chamber test (sociability) at P32–P48. Right, quantification of time spent sniffing the mouse (social) and empty (nonsocial) in Control and Sevo groups (Control: P < 0.001; Sevo: P < 0.001; Mann-Whitney test). (D) Quantification of spontaneous activity in Control and Sevo groups at P28–P31 (movements: P = 0.150; standings: P = 0.693; Mann-Whitney test). (E) The RPM taken for the mice to fall from the rod from Control and Sevo groups at P56 in Rotarod test (P = 0.632; Mann-Whitney test). (F) Number of entrances to the central area (left, P = 0.565; Mann-Whitney test), time spent in the central area (middle, P = 0.346; Mann-Whitney test), and total distance traveled (right, P = 0.893, unpaired t test) in the open field test in Control and Sevo groups at P35. *P < 0.05; ***P < 0.001; n.s., not significant. Data are shown as mean ± SEM. Underlying data are available in S1 Data. n.s., not significant; RPM, rotations per min; Sevo, sevoflurane. (TIF) [file pbio.3000086.s007.tif]

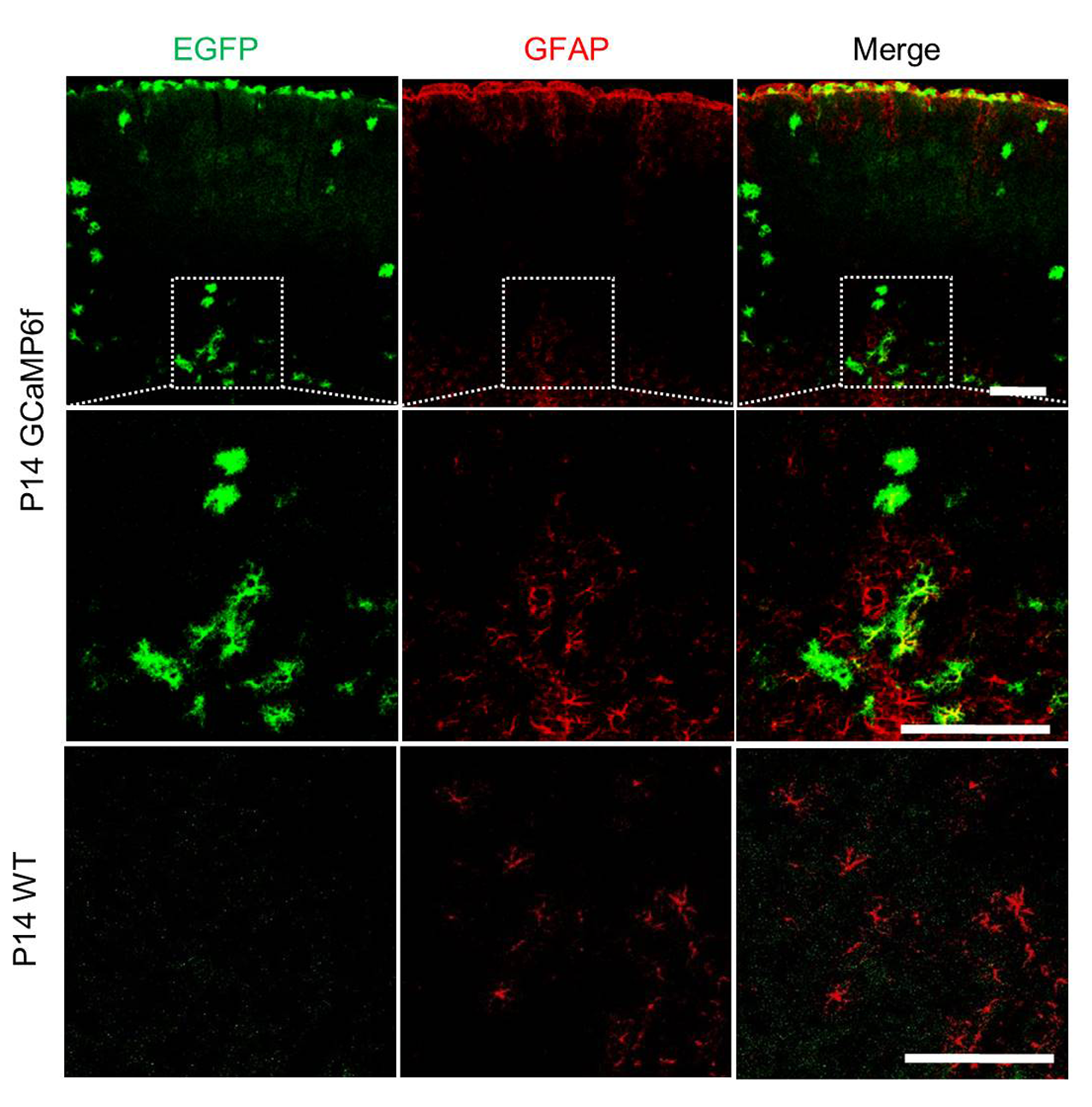

Supplement: S7 Fig — Fluorescent images of auto-EGFP (green) and GFAP (red) in the cortex of WT and GCaMP6f-injected mice at P14. Scale bars, 200 μm. EGFP, enhanced green fluorescent protein; GCaMP6f, AAV5•gfaABC1D•GCaMP6f; GFAP, glial fibrillary acidic protein; WT, wild-type. (TIF) [file pbio.3000086.s008.tif]

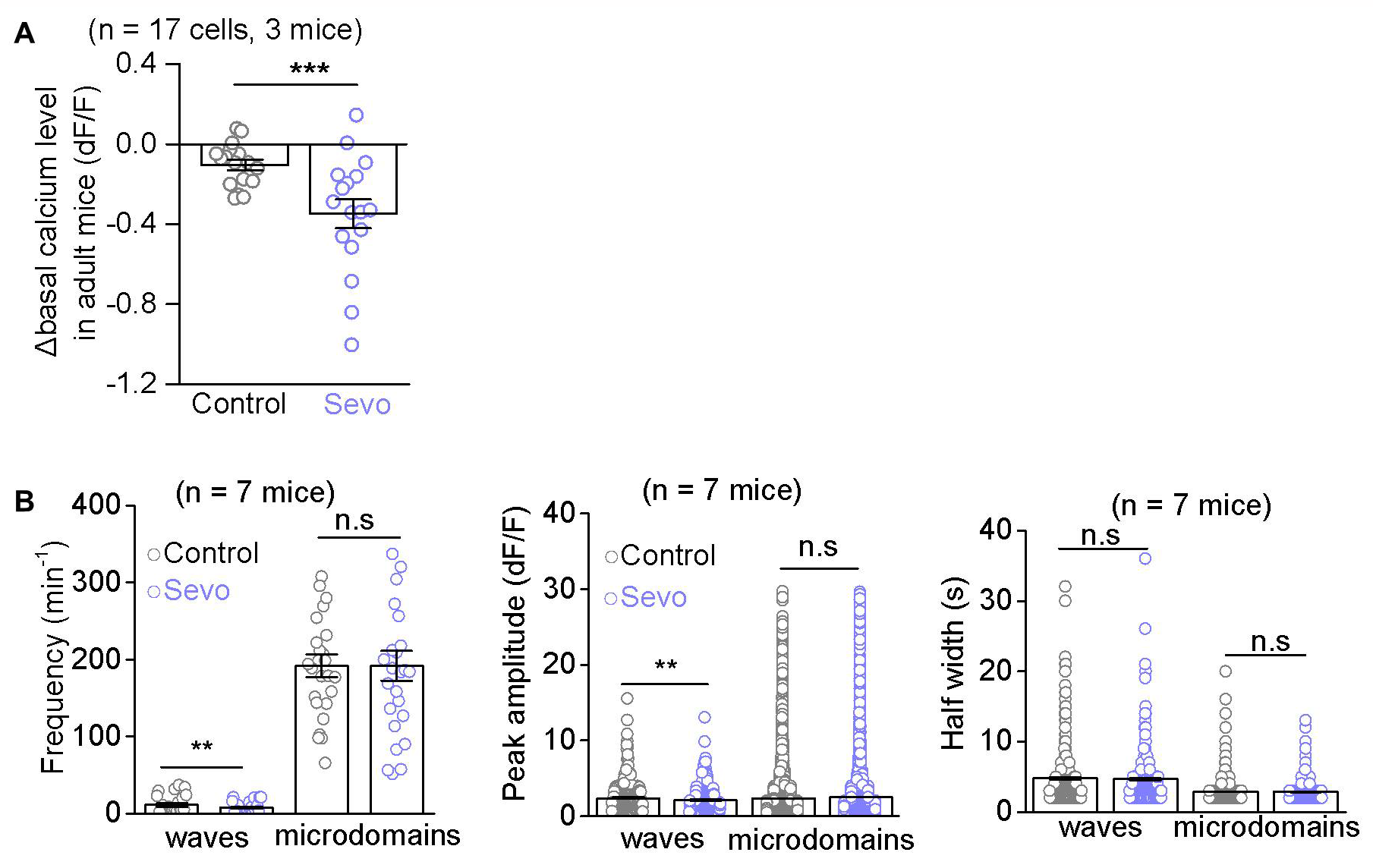

Supplement: S8 Fig — (A) Quantification of acute Sevo application on cortical astrocyte basal Ca2+ from adult mice at P56 (P = 0.004, unpaired t test). (B) Quantification of the two types of spontaneous Ca2+ signals properties, including frequency, peak amplitude, and half-width, before and after acute Sevo exposure (P = 0.009, paired t test, for waves frequency; P = 0.997, paired t test for microdomains frequency; P = 0.009, Mann-Whitney test for waves peak amplitude; P = 0.188, Mann-Whitney test for microdomains peak amplitude; P = 0.717, Mann-Whitney test for waves half-width; P = 0.742, Mann-Whitney test for microdomains half-width). Data are shown as mean ± SEM. Underlying data are available in S1 Data. n.s., not significant; Sevo, sevoflurane. (TIF) [file pbio.3000086.s009.tif]

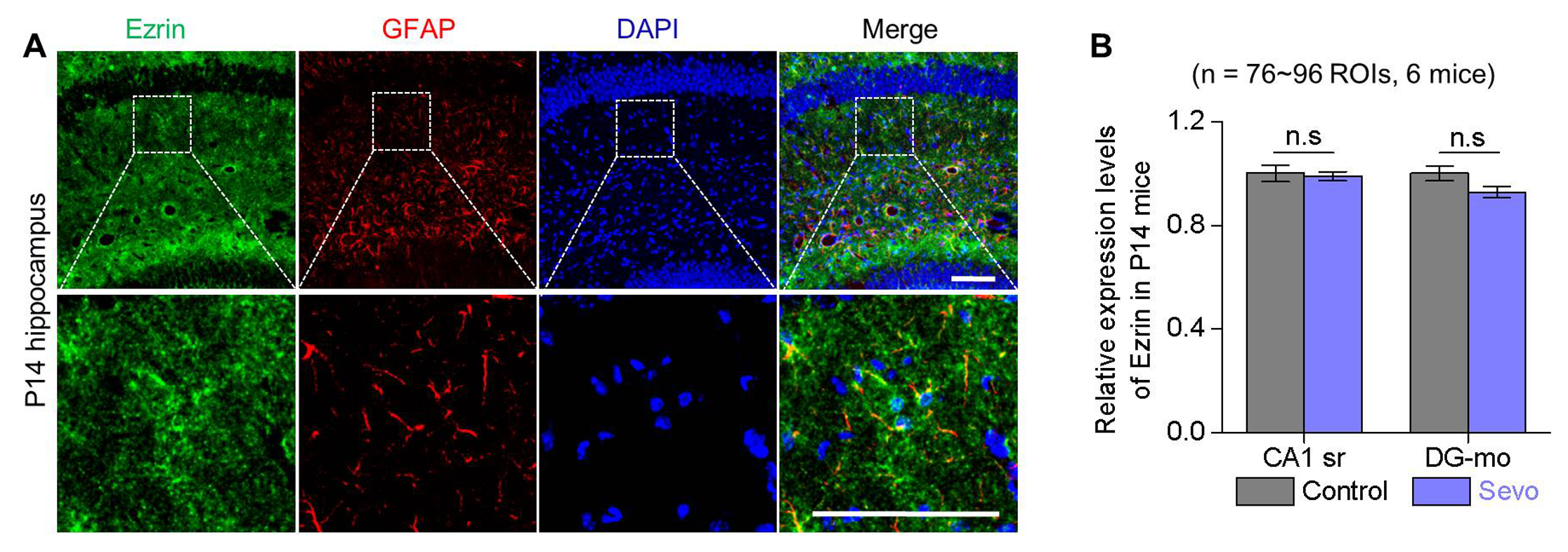

Supplement: S9 Fig — (A) Representative fluorescent images in the hippocampus of Control and Sevo group mice at P14. Scale bars, 200 μm. (B) Quantification of Ezrin fluorescent intensity in the hippocampal CA1sr (n = 96 ROIs from 6 mice in the Control group, n = 86 ROIs from 6 mice in the Sevo group; P = 0.295, Mann-Whitney test) and DG-mo (n = 84 ROIs from 6 mice in the Control group, n = 76 ROIs from 6 mice in the Sevo group; P = 0.164, Mann-Whitney test). Data are shown as mean ± SEM. Underlying data are available in S1 Data. CA1sr, CA1 stratum radiatum; DG-mo, molecular layer of dentate gyrus; n.s., not significant; ROI, region of interest. (TIF) [file pbio.3000086.s010.tif]

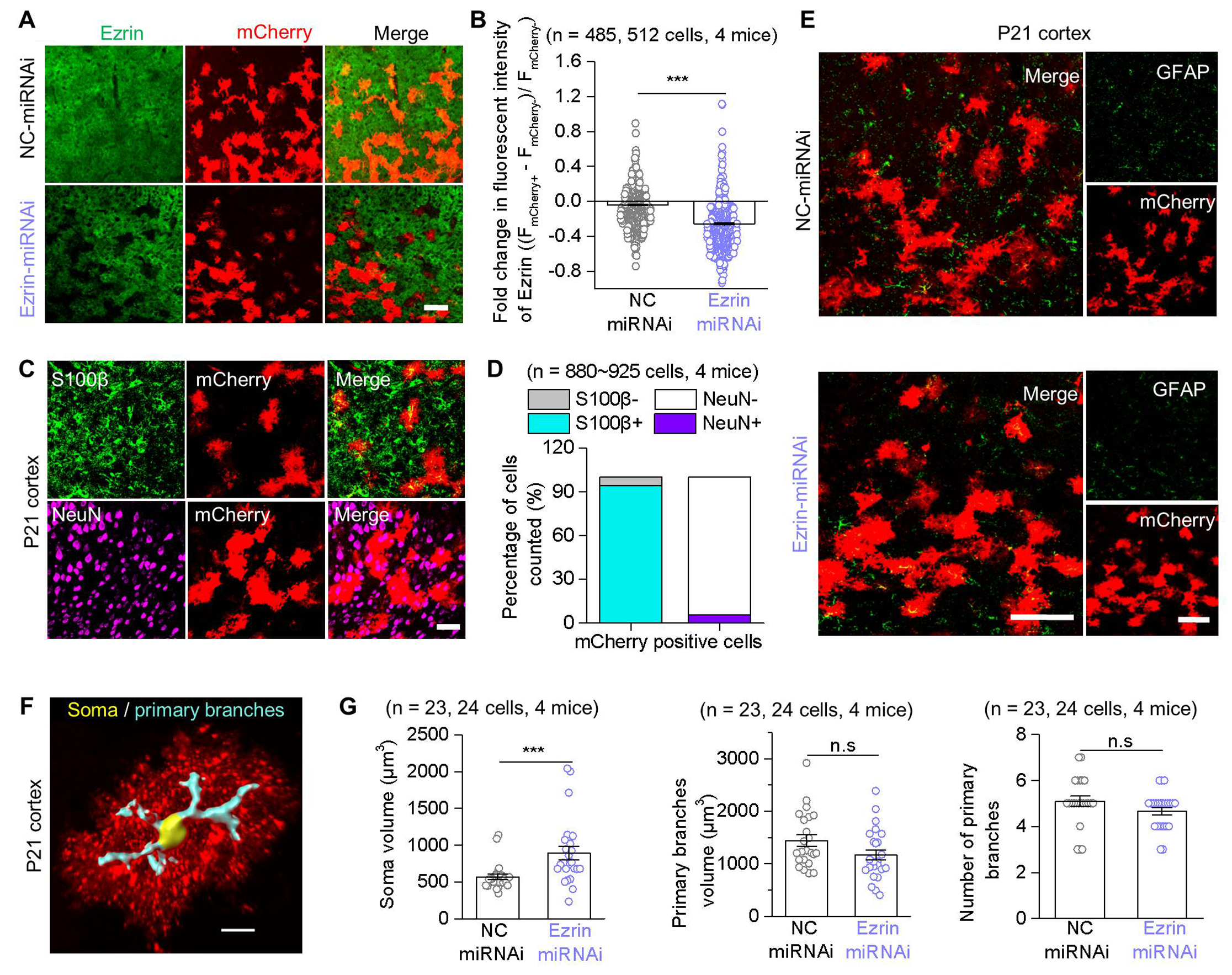

Supplement: S10 Fig — (A) Fluorescent images of Ezrin and mCherry in the cortex of NC-miRNAi and Ezrin-miRNAi mice at P21. Scale bars, 100 μm. (B) Quantification of fold change in Ezrin fluorescent intensity, measured as (FmCherry+ − FmCherry−)/ FmCherry− (P = 0, Mann-Whitney test). (C) Schematic fluorescent images of S100β, mCherry, and NeuN in the cortex of Ezrin-miRNAi–injected mice at P21. Scale bars, 50 μm. (D) Quantification of mCherry+ cells colocalized with S100β and NeuN. (E) Images of GFAP and mCherry in the cortex of Ezrin-miRNAi and NC-miRNAi–injected mice at P21. Scale bars, 20 μm. (F) Confocal image with 3D reconstructed soma and primary branches of mCherry-labeled astrocyte. Scale bar, 10 μm. (G) Quantification of astrocyte soma volume (left), primary branches volume (middle), and number of primary branches (right), respectively, in NC-miRNAi and Ezrin-miRNAi–injected mice (soma volume: P < 0.001, number of primary branches: P = 0.095, Mann-Whitney test; primary branches volume: P = 0.072, unpaired t test). ***P < 0.001; n.s., not significant. Data are shown as mean ± SEM. Underlying data are available in S1 Data. GFAP, glial fibrillary acidic protein; KD, knock-down; miRNAi, microRNA-based RNA interference; NC, negative control; NeuN, neuronal nuclei; n.s., not significant. (TIF) [file pbio.3000086.s011.tif]

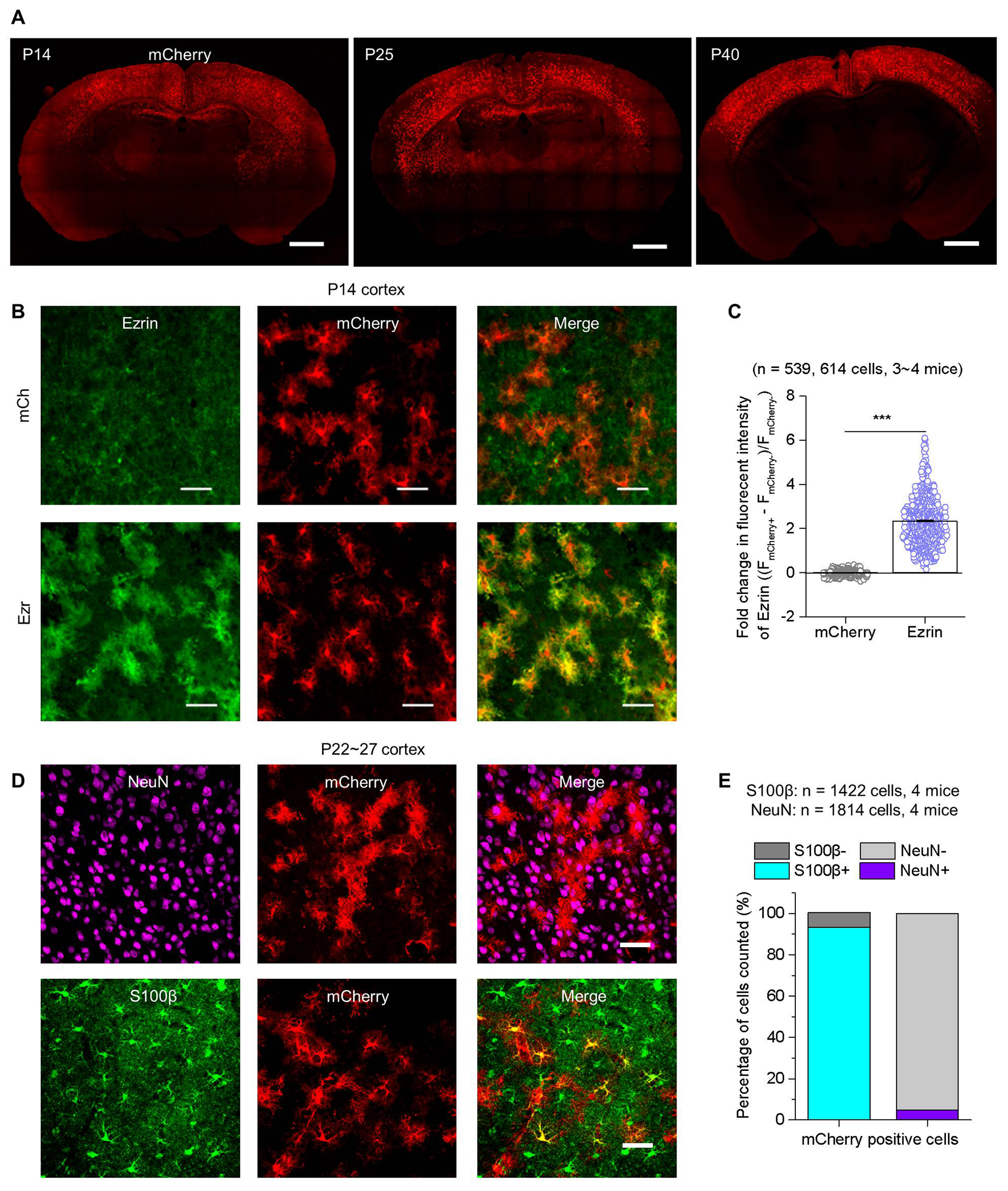

Supplement: S11 Fig — (A) The autofluorescence of mCherry showed that Ezr (AAV8·gfaABC1D·Ezrin·P2A·mCherry) was widely expressed in the brain, especially the cortex, of mice at P9, P25, and P40. Scale bars, 1,000 μm. (B) Fluorescent images of Ezrin staining (green) and mCherry (red) in the cortex of mCh (AAV8-gfaABC1D- mCherry) and Ezr-injected mice at P14. Scale bars, 50 μm. (C) Quantification of fold change in Ezrin fluorescent intensity, measured as (FmCherry+ − FmCherry−)/ FmCherry− (P < 0.001, Mann-Whitney test). (D) Fluorescent images of NeuN (magenta), mCherry (red), and S100β (green) in the cortex of Ezr-injected mice. Scale bars, 50 μm. (E) Quantification of mCherry+ cells colocalized with S100β and NeuN. ***P < 0.001. Data are shown as mean ± SEM. Underlying data are available in S1 Data. Ezr, AAV8•gfaABC1D•Ezrin•P2A•mCherry; mCh, AAV8•gfaABC1D•mCherry; NeuN, neuronal nuclei. (TIF) [file pbio.3000086.s012.tif]

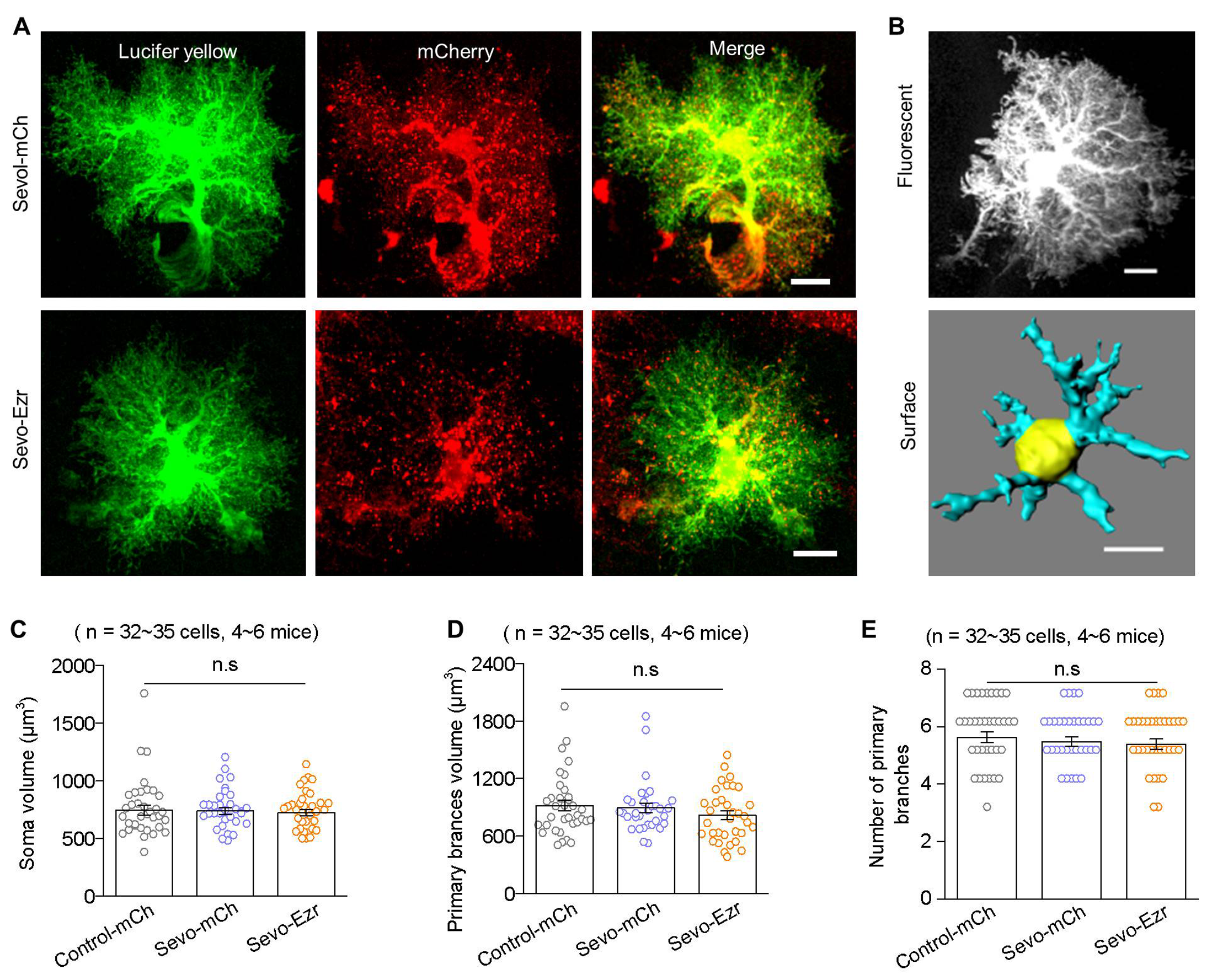

Supplement: S12 Fig — (A) Confocal images of astrocytes labeled with Lucifer yellow (green) and mCherry (red) in the cortex of mCh- and Ezr-injected mice at P14, in which mCherry was insufficient to mark astrocytic fine processes. Scale bars, 10 μm. (B) Representative confocal image, 3D reconstructed soma and primary branches of a cortical astrocyte. Scale bar, 10 μm. (C) Quantification of the soma volume of cortical astrocytes in mice from Control-mCh, Sevo-mCh, and Sevo-Ezr groups (P = 0.894, Kruskal-Wallis test). (D, E) Quantification of astrocytic primary branches volume (D) and number (E) in the cortex of mice from Control-mCh, Sevo-mCh, and Sevo-Ezr groups (volume: P = 0.385; number: P = 0.599; Kruskal-Wallis test). Data are shown as mean ± SEM. Underlying data are available in S1 Data. Ezr, AAV8•gfaABC1D•Ezrin•P2A•mCherry; mCh, AAV8•gfaABC1D•mCherry; n.s., not significant; Sevo, sevoflurane. (TIF) [file pbio.3000086.s013.tif]

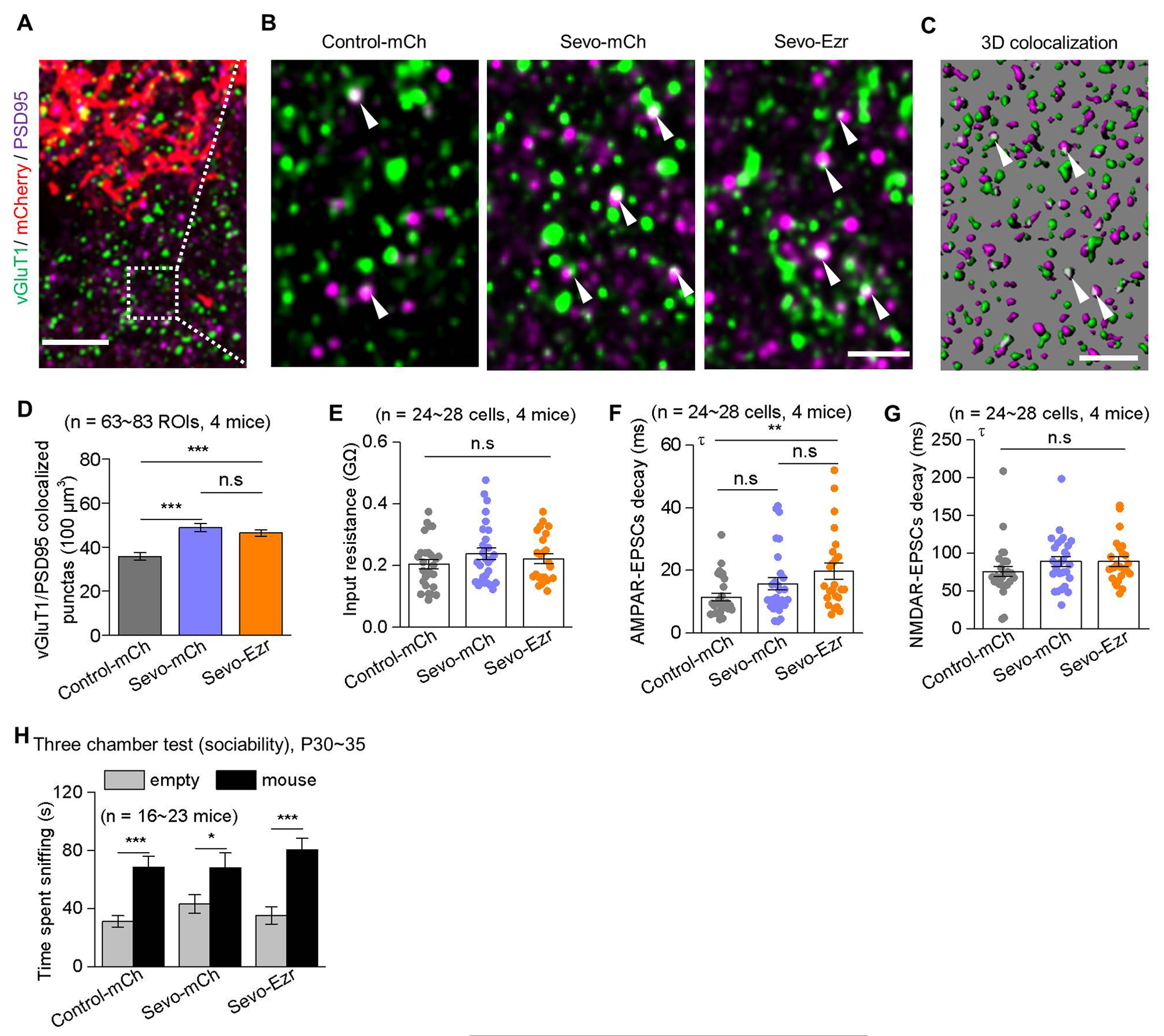

Supplement: S13 Fig — Characterization of the input resistance, decay kinetics of AMPAR/NMDAR-mediated eEPSCs, and sociability in the three groups of mice. (A) Fluorescent image of vGluT1 (green) and PSD95 (magenta) in mCherry-positive (mCherry+) and mCherry-negative (mCherry−) area. Scale bar, 5 μm. (B) Zoomed-in images of vGluT1 and PSD95 in mCherry− area from the cortex of Control-mCh, Sevo-mCh, and Sevo-Ezr groups. Scale bar, 2 μm. (C) Colocalization of vGluT1 and PSD95; the white arrows showed vGluT1/PSD95 colocalized puncta. Scale bar, 2 μm. (D) Quantification of vGluT1/PSD95 colocalized puncta in mCherry− area (Control-mCh versus Sevo-mCh: P < 0.001; Control-mCh versus Sevo-Ezr: P < 0.001; Sevo-mCh versus Sevo-Ezr: P = 0.511; Kruskal-Wallis test followed by post hoc multiple comparison test). (E) Input resistance of pyramidal neurons in Control-mCh, Sevo-mCh, and Sevo-Ezr groups (P = 0.379, one-way ANOVA). (F) Quantification of the decay kinetics (weighted time constants) of AMPAR-mediated eEPSCs in Control-mCh, Sevo-mCh, and Sevo-Ezr groups (Control-mCh versus Sevo-mCh: P = 0.099; Sevo-mCh versus Sevo-Ezr: P = 0.195; Control-mCh versus Sevo-Ezr: P = 0.004; Kruskal-Wallis test followed by post hoc multiple comparison test). (G) Quantification of the decay kinetics (weighted time constants) of NMDAR-mediated eEPSCs in Control-mCh, Sevo-mCh, and Sevo-Ezr groups (P = 0.124, Kruskal-Wallis test). (H) Quantification of time spent sniffing the mouse (social) and empty (nonsocial) in three-chamber sociability test at P30–P35 (Control-mCh: P < 0.001, paired t test; Sevo-mCh: P = 0.047, paired t test; Sevo-Ezr: P < 0.001, Mann-Whitney test). *P < 0.05; *** P < 0.001; n.s., not significant. Data are shown as mean ± SEM. Underlying data are available in S1 Data. AMPAR, α-amino-3-hydroxy-5-methyl-4-isoxazole propionate receptor; eEPSC, evoked excitatory postsynaptic current; Ezr, AAV8•gfaABC1D•Ezrin•P2A•mCherry; mCh, AAV8•gfaABC1D•mCherry; NMDAR, N-methyl-D-aspartic acid receptor; n.s., not s [file pbio.3000086.s014.tif]
